# Global Burden of Type 2 Diabetes Attributable to Behavioral Risks: Insights and Projections to 2050 Based on the Global Burden of Disease Study 2021

**DOI:** 10.3389/ijph.2026.1608765

**Published:** 2026-03-11

**Authors:** Guomiao Zhang, Huiqin Mei, Qichao Sheng, Yang Chen, Xinlv Zhang, Anthony Diwon, Hui Wang, Yuxin Chen, Ziyi Wang, Xiaoyu Zhang, Qingyang Mao, Dapeng Li, Chao Zheng, Guangyun Mao, Fang Shi

**Affiliations:** 1 Department of Epidemiology and Health Statistics, School of Public Health, Wenzhou Medical University, Wenzhou, Zhejiang, China; 2 State Key Laboratory of Cognitive Intelligence, University of Science and Technology of China, Hefei, Anhui, China; 3 Department of Endocrinology, The Second Affiliated Hospital, School of Medicine, Zhejiang University, Hangzhou, Zhejiang, China; 4 Department of Environmental Health Sciences, School of Public Health, University of Alberta, Edmonton, AB, Canada

**Keywords:** type 2 diabetes, behavioral risks, GBD 2021, prevention strategy, projection

## Abstract

**Objectives:**

This study aims to provide a comprehensive analysis of the burden of Type 2 diabetes (T2D) attributable to behavioral risks.

**Methods:**

Utilizing the Global Burden of Disease (GBD) 2021 data for secondary modeling, we analyzed the burden of T2D attributable to behavioral risks, stratified by age, gender, risk factors, and regions. A Bayesian age-period-cohort (BAPC) model projected burden trajectories from 2022 to 2050 under the continuation of historical trends.

**Results:**

From 1990 to 2021, global deaths and DALYs of T2D attributable to behavioral risks increased by 133.87% and 187.68%. The greatest rises in ASMR and ASDR occurred in Eastern Europe, Central Asia, and Southern Sub-Saharan Africa. Dietary risks remained the primary contributor, whereas the T2D burden attributable to high alcohol use exhibited the steepest increase from 1990 to 2021. The global ASMR and ASDR increased exponentially with age and were consistently higher in males. Projections from the BAPC model indicate that ASDR is expected to continue increasing through 2050.

**Conclusion:**

T2D burden attributable to behavioral risks is increasing rapidly, underscoring the need for targeted interventions and public health education.

## Introduction

Diabetes mellitus is a significant global health challenge, currently ranking as the 8th leading cause of death and disability worldwide [1]. Type 2 diabetes (T2D), which is primarily characterized by insulin resistance, accounts for 90%–95% of all diabetes cases and significantly contributes to global mortality rates [2]. In 2021, T2D and its complications were responsible for an estimated 6.7 million deaths among adults aged 20 to 79 [3]. The age-standardized global prevalence rate of T2D is projected to increase by 61.2%, rising from 5.9% in 2021 to 9.5% in 2050, potentially affecting over 1.27 billion individuals [4].

Available evidence has demonstrated a significant link between behavioral risks and health loss in individuals with T2D. Adopting a balanced diet [5, 6], engaging in regular physical activity [7], and quitting smoking [8] have each been independently linked to a reduced risk of developing T2D. Meta-analyses of prospective cohort studies have found that individuals who consume moderate amounts of alcohol have a 30% lower incidence of diabetes than non-drinkers [9, 10]. Evidence suggests that structured behavioral interventions, such as increased physical activity and dietary modifications (e.g., higher fiber intake, reduced total calories, fats, and sugar-sweetened beverages), can reduce the incidence of T2D by 30%–60% among prediabetic individuals [11]. Moreover, adopting preventive behaviors may decrease the incidence of diabetic retinopathy and cardiovascular mortality [12]. Therefore, understanding the burden of T2D attributable to behavioral risks and identifying these risks are crucial for developing targeted strategies to reduce the incidence and delay the progression of diabetes.

The Global Burden of Disease (GBD) Study 2021 offers a comprehensive analysis of the global, regional, and national burden of T2D attributable to various risk factors from 1990 to 2021. Among these, behavioral risks such as tobacco use, high alcohol consumption, dietary risks, and low physical activity have been increasingly recognized for their role in the development and progression of T2D [13]. As these behavioral risks are modifiable, health education at the individual level can support early prevention and delay the progression of T2D.

Although studies have reported on the global burden and trends in T2D from 1990 to 2021 [14], and on 88 risk factors in 204 countries and 811 subnational locations [15], there remains a gap in research specifically addressing the global burden and long-term trends of T2D attributable to behavioral risks. Using data from the GBD study 2021, this study aims to estimate the current global T2D burden associated with behavioral risks, predict its trends from 2022 to 2050, and explore differences in the burden across genders, ages and countries with varying socio-economic development levels. The findings may provide important evidence for the global targeted T2D prevention and control strategies.

## Methods

### Study Population and Data Collection

We conducted a global analysis of T2D attributable to behavioral risks using data from the Global Health Data Exchange query tool (GHDx, https://ghdx.healthdata.org/gbd-results-tool). Our metrics of interest included deaths, disability-adjusted life years (DALYs), the age-standardized mortality rate (ASMR), and the age-standardized disability-adjusted life years rate (ASDR), expressed per 100,000 people. The dataset covered various demographic groups across 204 countries, 21 global burden of disease regions, and 5 socio-demographic index (SDI) regions from 1990 to 2021. The SDI is a composite indicator reflecting a country’s development level. A higher SDI value, ranging from 0 to 1, indicates greater socio-economic development [16]. Countries and regions were classified into 5 SDI groups: High, High-middle, Middle, Low-middle, and Low SDI.

### Definition of T2D

T2D is a chronic condition occurring primarily in adults, characterized by insulin resistance or pancreatic insulin production deficiency. The GBD defines T2D as having a fasting plasma glucose level of ≥7 mmol/L (126 mg/dL) or currently taking drugs or insulin therapy. According to the International Classification of Diseases (ICD), Ninth Revision, the code directly assigned to T2D deaths is 250, and the Tenth Revision code is E11 [17].

### Estimation of Behavioral Risks

Data on behavioral risks were extracted from the GHDx following the definitions and classifications applied in the GBD 2021 study. The GBD study applies a hierarchical framework of risk factors, allowing both specific risks (e.g., Diet low in fruits, Diet low in vegetables) and aggregated categories (e.g., Dietary risks) to be evaluated simultaneously. The four behavioral risk categories for T2D included tobacco, high alcohol use, dietary risks, and low physical activity. In order to quantify the burden attributable to each behavioral risk, exposures were assessed relative to a theoretical minimum risk exposure level (TMREL)—the counterfactual level of exposure that would minimize the risk of diabetes, according to epidemiological evidence [4, 15]. On this basis, tobacco included both active smoking and exposure to secondhand smoke, with the TMREL defined as lifelong non-use. High alcohol use was defined as alcohol consumption in excess of the TMREL, the level of alcohol consumption at which all-cause risk is minimized [18]. However, the contribution of each risk factor to overall health loss varies by geography, age, period, and gender, indicating that the amount of alcohol that minimizes health loss also differs across these dimensions (Supplementary Appendix S3). Dietary risks were subdivided into 7 components, each with a TMREL defined according to the GBD 2021 criteria. Protective factors included intake above the TMREL, such as fruits (≥340–350 g/day), vegetables (≥306–372 g/day), whole grains (≥160–210 g/day), and dietary fibre (≥22–25 g/day). Harmful factors were defined as intake below the TMREL, including processed meat (0 g/day) and sugar-sweetened beverages (0 g/day). A J-shaped association was observed between red meat intake and T2D, with the lowest risk occurring at 0–200 g/day. Low physical activity was assessed using metabolic equivalent minutes per week (MET-min/week) across leisure, occupational, household, and transport domains. The TMREL was set at 3,600–4,400 MET-min/week, corresponding to the level associated with the lowest risk of adverse outcomes. Detailed descriptions of the risk factor estimation methodology are provided in Supplementary Appendix S4.

### Statistical Analysis

To appropriately measure the burden of T2D, we utilized the count of deaths, DALYs, ASMR, and ASDR attributed to behavioral risks with 95% uncertainty intervals (95% UIs). The 95% UI refers to the 2.5th and 97.5th percentiles of the 1,000 draw-level estimates for each parameter [19]. Countries and territories were divided into 21 GBD regions based on geographical similarities and were grouped into 5 SDI regions depending on their SDI levels. Age-standardized rates (ASR) were used to estimate the T2D burden attributable to behavioral risks at the global, regional, national, and territorial levels in 1990 and 2021. We calculated the Average Annual Percentage Changes (AAPCs) using the Joinpoint regression model, where the number of joinpoints was determined by the Permutation Test with a default maximum number of three [20]. The model calculations are as follows:

$$\ln\left(y\right)=\alpha +\beta_{i}x+\varepsilon$$

$$Y=\left[\exp\frac{\sum w_{i}\beta_{i}}{\sum w_{i}}-1\right]\times 100$$

In the above equations, y represented ASMR or ASDR, x was the calendar year, β_i_ indicated the slope coefficients of each segment in the expected year range, Y referred to AAPCs and w_i_ was the length of each segment in the year range [21]. An increasing trend in ASMR or ASDR was indicated when both the AAPCs and its 95% confidence interval (95% CI) were greater than 0, and a decreasing trend when both were below zero. If the 95% CIs of AAPCs included 0, the ASMR and ASDR were considered stable.

Necessary stratified analyses by age and gender were additionally employed to identify sensitive populations for T2D attributable to behavioral risks. Restricted cubic spline (RCS) regression models were further performed to explore changes of ASMR and ASDR in 21 regions with different SDI levels from 1990 to 2021. The association of AAPCs with SDI in 2021 for ASMR and ASDR at the national level was further examined using locally estimated scatterplot smoothing (LOESS) regression.

To project future trends in T2D-related deaths and DALYs up to 2050, we utilized Bayesian age-period-cohort (BAPC) analysis, a widely applied approach in epidemiology for forecasting disease burden based on historical patterns. The BAPC model integrates both prior knowledge and observed data to provide probabilistic forecasts of disease burden, with particular attention to uncertainties [22]. Using data from 1990 to 2021, we trained the model and produced forecasts for 2050, with separate trend analyses for males and females. Uncertainty intervals were calculated using Bayesian inference, providing robust estimates for future trends [23, 24].

Data management and analyses were performed using Joinpoint Regression Program (version 5.2.0) and R Statistical Software (version 4.4.1).

## Results

### Global Trends in T2D Burden Attributable to Behavioral Risks From 1990 to 2021

Globally, deaths of T2D attributable to behavioral risks grew significantly (AAPCs = 2.75, 95% CI: 2.58−2.92, P < 0.001), increasing from approximately 267,955 cases in 1990 to 626,661 cases in 2021, while the corresponding ASMR did not change much, with AAPCs of 0.06 (95% CI: −0.06−0.17, P > 0.05). Furthermore, the DALYs of T2D attributable to behavioral risks also showed a substantial rise (AAPC = 3.47, 95% CI: 3.40−3.55, P < 0.001), increasing from 10.49 million in 1990 to 30.19 million in 2021. In addition, the ASDR of T2D increased from 260.56 in 1990 to 348.52 per 100,000 people in 2021, with AAPCs of 0.96 (95% CI: 0.92−1.00, P < 0.001) (Table 1; Supplementary Table S1).

**TABLE 1:** The age-standardized mortality rate and age-standardized disability-adjusted life years rate of Type 2 diabetes attributable to behavioral risks in 1990 and 2021 at the global and regional level, and their Average Annual Percentage Changes from 1990 to 2021 (Global Burden of Disease Study, 21 Global Burden of Disease regions, 1990–2021).

Region	Age-standardized rate per 100,000 people (95% UI)				Average annual percentage changes (AAPCs) from 1990 to 2021 (95% CI)	
	1990		2021			
	Death	DALYs	Death	DALYs	Deaths rate	DALYs rate
Global	7.40 (4.40,9.63)	260.56 (147.88,350.55)	7.42 (4.36,9.75)	348.52 (192.6,486.92)	0.06 (−0.06,0.17)	0.96 (0.92,1.00)^***^
SDI category						
High SDI	6.36 (3.73,8.27)	240.22 (138.35,322.50)	4.43 (2.47,5.83)	338.88 (179.05,489.92)	−1.17 (−1.40, −0.94)^***^	1.11 (1.06,1.17)^***^
High-middle SDI	6.01 (3.63,7.68)	229.04 (132.26,305.75)	5.55 (3.30,7.31)	296.49 (166.59,413.4)	−0.26 (−0.54,0.02)	0.87 (0.78,0.96)^***^
Middle SDI	7.70 (5.08,9.83)	270.48 (169.47,360.66)	8.26 (5.12,10.73)	348.93 (207.63,483.24)	0.26 (0.05,0.47)^*^	0.83 (0.77,0.90)^***^
Low-middle SDI	9.59 (5.61,12.83)	294.34 (170.88,394.29)	12.30 (7.22,16.18)	428.62 (245.72,591.23)	0.82 (0.48,1.17)^***^	1.25 (1.16,1.34)^***^
Low SDI	12.35 (5.46,17.78)	356.63 (155.58,515.83)	12.32 (5.78,17.51)	405.19 (178.58,588.02)	0.02 (−0.1,0.14)	0.41 (0.36,0.46)^***^
Southeast Asia, East Asia, and Oceania Region	5.32 (3.60,6.82)	208.87 (133.76,276.57)	5.28 (3.48,7.02)	269.66 (163.88,377.05)	−0.01 (−0.12,0.11)	0.84 (0.75,0.93)^***^
East Asia	3.93 (2.62,5.15)	179.28 (111.10,245.21)	3.78 (2.41,5.29)	240.22 (138.68,345.19)	−0.07 (−0.27,0.13)	0.98 (0.83,1.14)^***^
Southeast Asia	9.45 (6.29,12.25)	1,200.33 (668.94,1703.97)	10.08 (6.9,13.12)	368.75 (243.03,489.65)	0.21 (0.15,0.28)^***^	0.69 (0.62,0.75)^***^
Oceania	41.46 (22.48,59.48)	296.96 (189.13,384.59)	42.72 (23.4,58.4)	1,418.2 (783.5,1946.81)	0.08 (0.04,0.13)^***^	0.52 (0.48,0.57)^***^
Central Europe, Eastern Europe, and Central Asia Region	3.59 (1.95,4.70)	192.26 (100.60,263.14)	6.59 (3.54,8.74)	333.81 (172.99,464.38)	2.01 (1.23,2.79)^***^	1.81 (1.47,2.14)^***^
Central Asia	4.57 (2.20,6.07)	213.81 (103.95,299.22)	8.30 (4.00,11.45)	431.71 (205.40,611.84)	1.95 (1.61,2.29)^***^	2.25 (2.02,2.49)^**^
Central Europe	6.24 (3.70,8.03)	288.81 (166.32,388.98)	6.74 (3.97,8.88)	370.15 (208.19,510.70)	0.31 (−0.07,0.70)	0.84 (0.65,1.04)^***^
Eastern Europe	1.99 (1.01,2.65)	135.83 (65.38,191.87)	5.88 (3.06,7.80)	278.66 (141.11,387.50)	3.57 (2.77,4.37)^***^	2.37 (2.10,2.64)^***^
High-income Region	7.01 (4.09,9.09)	249.92 (142.92,333.86)	4.36 (2.40,5.73)	329.75 (171.12,478.18)	−1.51 (−1.67,-1.35)^***^	0.89 (0.85,0.94)^***^
High-income Asia Pacific	3.98 (2.60,5.05)	215.47 (134.47,289.12)	1.61 (0.96,2.14)	278.40 (149.26,417.13)	−0.77 (−0.83,-0.71)^***^	0.76 (0.57,0.94)^***^
Australasia	5.86 (3.32,7.73)	198.73 (109.99,270.37)	4.30 (2.34,5.81)	226.83 (114.23,329.02)	−0.91 (−1.61,-0.21)^*^	0.47 (0.08,0.87)
Western Europe	7.46 (4.42,9.66)	230.68 (132.43,307.00)	4.20 (2.35,5.54)	257.73 (135.83,373.81)	−1.80 (−1.94,-1.66)^***^	0.35 (0.27,0.43)^***^
Southern Latin America	11.31 (5.97,15.03)	357.59 (187.90,484.87)	7.61 (3.84,10.16)	396.00 (195.75,578.06)	−1.37 (−1.82,-0.92)^***^	0.41 (0.10,0.71)^**^
High-income North America	7.33 (4.07,9.57)	287.58 (159.52,390.46)	5.99 (3.21,7.88)	458.82 (236.46,662.81)	−0.63 (−0.86,-0.39)^***^	1.53 (1.40,1.65)^***^
Latin America and Caribbean Region	14.39 (8.98,18.61)	488.70 (292.06,658.81)	12.53 (7.07,17.32)	504.25 (264.33,724.80)	−0.40 (−0.75,-0.06)^*^	0.11 (−0.11,0.34)
Caribbean	16.51 (9.46,22.14)	539.22 (299.25,740.54)	12.67 (7.02,17.81)	582.15 (315.24,816.18)	−0.91 (−1.61,-0.21)^***^	0.28 (0.10,0.46)^*^
Andean Latin America	6.15 (3.30,8.69)	204.16 (102.39,292.87)	7.10 (3.69,10.43)	289.75 (145.30,430.76)	0.26 (0.05,0.47)^**^	1.17 (1.01,1.32)^***^
Central Latin America	16.76 (10.69,21.85)	575.90 (341.16,782.17)	15.34 (8.32,21.56)	603.82 (315.90,871.72)	−0.32 (−0.65,0.01)	0.16 (−0.28,0.61)
Tropical Latin America	13.54 (8.89,17.35)	458.54 (293.61,598.56)	11.00 (6.36,14.70)	439.39 (233.35,624.46)	−0.66 (−1.09,-0.23)^**^	−0.11 (−0.36,0.13)
North Africa and Middle East Region	10.09 (6.65,13.13)	329.04 (201.72,440.92)	11.92 (7.42,15.53)	549.62 (330.72,755.36)	0.58 (0.45,0.72)^***^	1.66 (1.61,1.71)^***^
South Asia Region	9.22 (5.57,12.40)	277.59 (162.97,371.59)	11.72 (6.89,15.78)	388.75 (220.16,538.70)	0.89 (0.58,1.19)^***^	1.11 (1.00,1.21)^***^
Sub-Saharan Africa Region	14.3 (6.45,20.41)	389.59 (169.70,562.29)	15.71 (7.46,22.03)	459.01 (209.98,653.69)	0.32 (0.24,0.41)^***^	0.53 (0.46,0.60)^***^
Central Sub-Saharan Africa	18.2 (6.83,28.03)	489.77 (172.45,743.07)	17.88 (6.06,27.87)	547.58 (182.01,837.15)	−0.08 (−0.2,0.05)	0.38 (0.30,0.46)^***^
Eastern Sub-Saharan Africa	16.06 (5.63,23.97)	425.82 (148.02,632.82)	12.70 (5.14,18.91)	354.88 (138.06,523.69)	−0.77 (−0.83,-0.71)^***^	−0.62 (−0.69,-0.56)^***^
Southern Sub-Saharan Africa	18.57 (11.3,24.37)	517.99 (307.12,685.71)	31.02 (17.42,41.44)	865.09 (479.81,1163.58)	1.74 (1.14,2.35)^***^	1.73 (1.25,2.22)^***^
Western Sub-Saharan Africa	10.50 (5.04,14.97)	292.43 (135.59,415.94)	12.80 (6.25,17.91)	398.15 (185.64,577.07)	0.64 (0.59,0.69)^***^	1.00 (0.95,1.04)^***^

*P < 0.05, **P < 0.01, ***P < 0.001.

In terms of 21 GBD regions, Southern Sub-Saharan Africa recorded the highest ASMR (31.02 per 100,000 people, 95% UI: 17.42–41.44) and ASDR (865.09, 95% UI: 479.81−1,163.58 per 100,000 people). High-income North America had a lower ASMR (5.99 per 100,000 people, 95% UI: 3.21–7.88) but still bore a high ASDR burden (458.82 per 100,000 people, 95% UI: 236.46–662.81). Eastern Europe showed the greatest increases in both ASMR (AAPCs = 3.57, 95% CI: 2.77−4.37, P < 0.001) and ASDR (AAPCs = 2.37, 95% CI: 2.10−2.64, P < 0.001) (Table 1).

**FIGURE 1:**
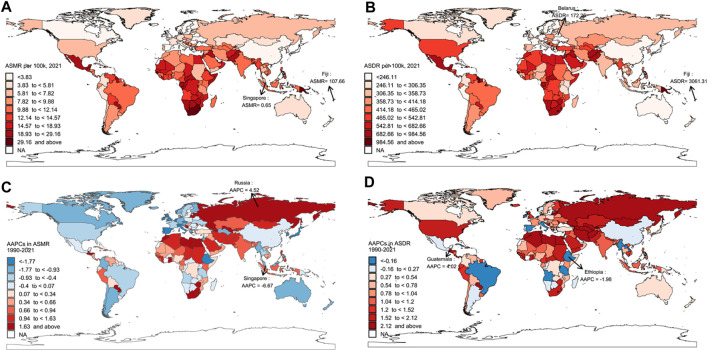
The age-standardized mortality rate and age-standardized disability-adjusted life years rate of Type 2 diabetes attributable to behavioral risks in 2021, and their Average Annual Percentage Changes from 1990 to 2021. **(A)** Age-standardized mortality rate; **(B)** Age-standardized disability-adjusted life years rate; **(C)** Average Annual Percentage Changes in age-standardized mortality rate; **(D)** Average Annual Percentage Changes in age-standardized disability-adjusted life years rate. ASMR, age-standardized mortality rate; ASDR, age-standardized disability-adjusted life years rate, AAPCs, Average Annual Percentage Changes (Global Burden of Disease Study, 204 countries or territories, 2021).

At the national level, Fiji had the highest ASMR (107.66 per 100,000 people) and ASDR (3,061.31 per 100,000 people) in 2021 (Figures 1A,B). Russia had the fastest ASMR increase (AAPC = 4.52), while Guatemala showed the largest increase in ASDR (AAPC = 4.02) (Figures 1C,D). India had the highest number of deaths, reaching 109,041.84, while China recorded the highest DALYs, amounting to 4,875,274.21 (Supplementary Figure S1).

### Global Trends in T2D Burden Attributable to Behavioral Risks by Region

**FIGURE 2:**
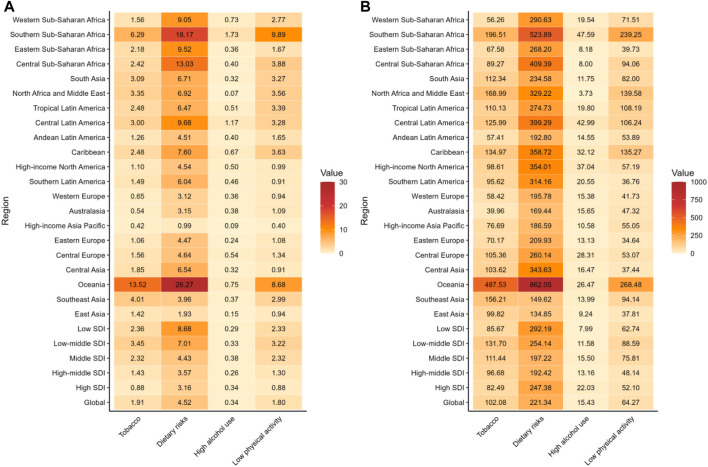
The age-standardized mortality rate and age-standardized disability-adjusted life years rate of Type 2 diabetes attributed to four behavioral risks in 2021. **(A)** Age-standardized mortality rate per 100,000 people; **(B)** Age-standardized disability-adjusted life years rate per 100,000 people (Global Burden of Disease Study, 21 Global Burden of Disease regions, 2021).

Our analysis revealed dietary risks were the predominant modifiable factors associated with T2D globally, with ASMR of 4.52 (95% UI: 0.88−7.36) and ASDR of 221.34 (95% UI: 47.97–368.92) per 100,000 people (Table 2). Specifically, the highest ASMR related to dietary risks was observed in Oceania (26.27 per 100,000 people, 95% UI: 4.25–44.14). Similarly, the highest ASDR was found in Oceania (862.05 per 100,000 people, 95% UI: 154.31–1,465.63) (Figure 2; Supplementary Table S2). Dietary risks, which encompassed seven secondary behavioral risks in the GBD study, showed significant regional variations in their impact on T2D. Globally, high consumption of processed meat, along with low intake of whole grains, were the top three dietary risk factors (Supplementary Figure S2).

**TABLE 2:** The global age-standardized mortality rate and age-standardized disability-adjusted life years rate of Type 2 diabetes attributable to behavioral risks in 1990 and 2021, and their Average Annual Percentage Changes from 1990 to 2021 (Global Burden of Disease Study, 1990–2021).

Behavioral risks	Age-standardized rate per 100,000 people (95% UI)				Average annual percentage changes (AAPCs) from 1990 to 2021 (95% CI)	
	1990		2021			
	Death	DALYs	Death	DALYs	Deaths rate	DALYs rate
Tobacco	2.21 (1.43,2.94)	90.76 (59.37,123.29)	1.91 (1.20,2.62)	102.08 (64.15,146.20)	−0.45 (−0.53, −0.38)^***^	0.38 (0.34,0.41)^***^
Smoking	1.34 (1.10,1.56)	57.55 (46.32,70.40)	1.08 (0.89,1.31)	62.48 (48.93,80.69)	−0.62 (−0.71, −0.53)^***^	0.27 (0.23,0.30)^***^
Secondhand smoke	0.96 (0.35,1.55)	36.33 (13.08,61.04)	0.87 (0.32,1.44)	42.52 (15.35,72.77)	−0.27 (−0.34, −0.20)^***^	0.50 (0.46,0.55)^***^
Dietary risks	4.55 (0.86,7.35)	159.96 (31.17,262.82)	4.52 (0.88,7.36)	221.34 (47.97,368.92)	0.03 (−0.10,0.15)	1.07 (1.03,1.12)^***^
Diet low in fruits	0.90 (0.14,1.58)	33.53 (5.21,60.47)	0.91 (0.14,1.59)	39.14 (6.17,70.26)	0.04 (−0.10,0.17)	0.51 (0.44,0.58)^***^
Diet low in vegetables	0.32 (−0.12,0.67)	9.73 (−3.71,20.60)	0.19 (−0.07,0.42)	6.87 (−2.66,15.21)	−1.64 (−1.78, −1.50)^***^	−1.15 (−1.23, −1.06)^***^
Diet low in whole grains	1.26 (0.36,2.05)	44.26 (13.14,74.13)	1.21 (0.33,1.98)	58.78 (17.32,98.86)	−0.07 (−0.16,0.02)	0.93 (0.90,0.96)^***^
Diet high in red meat	0.80 (−0.11,1.76)	28.96 (−4.39,64.77)	0.82 (−0.12,1.81)	44.10 (−6.73,100.38)	0.12 (−0.06,0.31)	1.39 (1.33,1.45)^***^
Diet high in processed meat	1.50 (0.36,2.45)	50.84 (12.44,85.40)	1.37 (0.32,2.27)	70.58 (17.11,120.71)	−0.32 (−0.52, −0.12)^**^	1.06 (0.97,1.15)^***^
Diet high in sugar-sweetened beverages	0.49 (0.25,0.70)	17.76 (9.18,26.50)	0.64 (0.33,0.93)	35.02 (17.59,53.13)	0.87 (0.75,0.99)^***^	2.22 (2.14,2.30)^***^
Diet low in fiber	0.24 (0.14,0.34)	8.31 (4.74,12.08)	0.21 (0.12,0.30)	9.02 (4.97,13.46)	−0.52 (−0.61, −0.43)^***^	0.24 (0.20,0.29)^***^
High alcohol use	0.29 (0.10,0.54)	8.89 (1.29,18.74)	0.34 (0.12,0.64)	15.43 (3.67,32.25)	0.57 (0.26,0.88)^***^	1.83 (1.68,1.97)^***^
Low physical activity	1.64 (0.71,2.51)	46.06 (19.90,70.69)	1.80 (0.79,2.75)	64.27 (28.01,100.49)	0.30 (0.16,0.43)^***^	1.10 (1.06,1.13)^***^

*P < 0.05, **P < 0.01, ***P < 0.001.

Tobacco emerged as the second leading behavioral risk factor for T2D, with a global ASMR of 1.91 and ASDR of 102.08 per 100,000 people (Table 2). The highest ASMR (13.52 per 100,000 people, 95% UI: 7.63–19.49) and ASDR (487.53 per 100,000 people, 95% UI: 290.44–695.67) for T2D was observed in Oceania (Figure 2; Supplementary Table S2). The risks of tobacco are made up of two main components, smoking and secondhand smoke. These factors showed similar geographic patterns, with the highest ASMR and ASDR in Oceania, Southern Sub-Saharan Africa, Caribbean and South Asia (Supplementary Figure S2).

While the overall burden of T2D attributable to high alcohol use was comparatively lower, there existed a notable increase in ASDR (AAPCs = 1.83, 95% CI: 1.68−1.97, P < 0.001) (Table 2). Southern Sub-Saharan Africa (1.73 per 100,000 people, 95% UI: 0.62–3.29) exhibited the highest ASMR for T2D linked to excessive alcohol intake. When considering ASDR, Southern Sub-Saharan Africa (47.59 per 100,000 people, 95% UI: 14.51–94.26), Central Latin America (42.99 per 100,000 people, 95% UI: 8.48–84.04), and High-Income North America (37.04 per 100,000 people, 95% UI: 7.55–74.61) were detected as the regions most affected by alcohol-related T2D (Figure 2; Supplementary Table S2).

Low physical activity was another key risk factor. Southern Sub-Saharan Africa had the highest ASMR (9.89 per 100,000 people, 95% UI: 4.18–15.08) and ASDR (239.25 per 100,000 people, 95% UI:102.53–361.94), followed closely by Oceania (ASMR: 8.68 per 100,000 people, 95% UI: 3.61−13.94; ASDR: 268.48 per 100,000 people, 95% UI:110.53–431.69) (Figure 2; Supplementary Table S2).

### Global Trends in T2D Attributable to Behavioral Risks by Gender and Age in 2021

**FIGURE 3:**
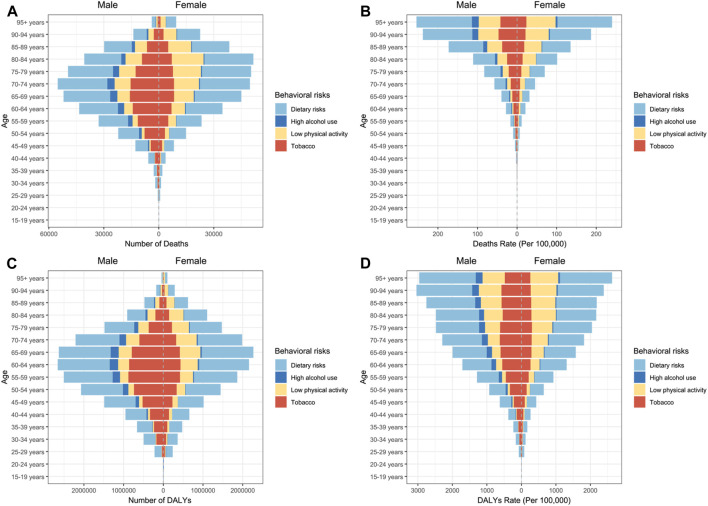
The number and rate of global burden of Type 2 diabetes attributable to behavioral risks by gender, age and risks in 2021. **(A)** Death numbers; **(B)** Deaths rate; **(C)** disability-adjusted life years; **(D)** disability-adjusted life years rate (Global Burden of Disease Study, 2021).

In 2021, T2D deaths attributable to behavioral risks varied by age, peaking in males aged 70–74 and females aged 80–84 (Figure 3A; Supplementary Table S3). The age-specific mortality rate increased exponentially with age, with higher rates observed in males than in females from age 30 onward (Figure 3B; Supplementary Table S4). The age groups most affected by DALYs due to T2D were males at ages 60–64 and females at ages 65–69, with total DALYs of 16.12 million for males and 14.07 million for females (Figure 3C; Supplementary Table S3). Consistent with the age-specific mortality rate, males showed higher age-specific DALYs rate than females, with rates increased substantially with age, except for males in the 90–94 age group (Figure 3D).

Gender differences in major behavioral risks were evident: males had higher exposure to tobacco and high alcohol use, while females showed lower levels of physical activity (Figure 3; Supplementary Table S5). In contrast, dietary risks were identified as the leading behavioral risk factor for T2D in both genders.

### Differences in T2D Burden Attributable to Behavioral Risks Across SDI Levels

From 1990 to 2021, deaths and DALYs of T2D attributable to behavioral risks increased globally and across five SDI regions, highest in middle SDI regions (Supplementary Figures S3A, B). Low-middle and low SDI regions had higher ASMRs and ASDRs than the global average (Supplementary Figures S4A, B).

In the 21 GBD regions, T2D deaths and DALYs from behavioral risks followed an inverted U-shaped trend with SDI, peaking at 0.59 before declining (Supplementary Figures S5A, B). It is worth noting that Southern Sub-Saharan Africa and Oceania had very high ASMR, with a significant increase between 1990 and 2021 (Supplementary Figures S6A). Oceania had a particularly high ASDR of 1,418.20 (95% UI: 783.5–1946.81) per 100,000 in 2021 and demonstrated a rapid upward trend (AAPC = 0.52, 95% CI: 0.48–0.57), surpassing regions with similar SDI (Supplementary Figure S6B; Table 1).

At the national level, a negative correlation between AAPCs and SDI was observed (ASMR: r = −0.503, P < 0.001; ASDR: r = −0.274, P = 0.002) when SDI was ≥0.53. However, the association weakened when the SDI fell below 0.53 (ASMR: r = 0.102, P = 0.470; ASDR: r = 0.105, P = 0.458) (Supplementary Figure S7).

### BAPC Prediction of T2D Burden Attributable to Behavioral Risks

**FIGURE 4:**
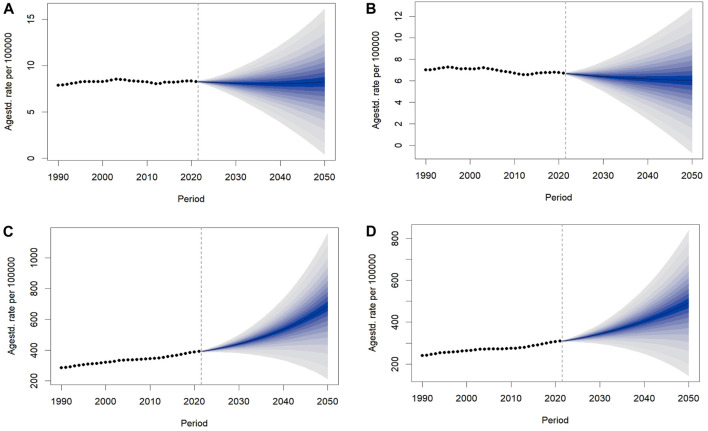
The trends of age-standardized rates of Type 2 diabetes attributable to behavioral risks worldwide from 2022 to 2050 predicted by Bayesian age-period-cohort model. **(A)** Projected age-standardized mortality rate per 100,000 people for males. **(B)** Projected age-standardized mortality rate per 100,000 people for females. **(C)** Projected age-standardized disability-adjusted life years rate per 100,000 people for males. **(D)** Projected age-standardized disability-adjusted life years rate per 100,000 people for females. ASMR, age-standardized mortality rate; ASDR, age-standardized disability-adjusted life years rate (Global Burden of Disease Study, 1990–2021).

Over the next 30 years, the projections indicate that the ASMR for both males and females will remain relatively stable (Figures 4A,B). In contrast, the projected ASDR has a substantial increase for both genders, with a more pronounced rise in males. Specifically, the ASDR of T2D attributable to behavioral risks will increase from 392.30 (95% CI: 392.11–392.49) in 2021 to 684.87 (95% CI: 207.38–1,162.37) per 100,000 people by 2050 in males, and from 309.80 (95% CI: 309.64–309.96) to 493.27 (95% CI: 191.90–794.63) per 100,000 people in females (Figures 4C,D). We also projected the global deaths and DALYs from 2022 to 2050. By 2050, the number of DALYs is expected to increase to 32.71 million in males and 23.56 million in females, highlighting a substantial disease burden (Supplementary Figure S8).

## Discussion

This study presents an in-depth analysis of the global burden of T2D attributable to behavioral risks, revealing the predominant behavioral risks across genders, age groups, and regions with diverse socioeconomic development statuses. The deaths and DALYs of T2D attributable to behavioral risks have doubled over the past 30 years, and BAPC projections indicate that ASDR will continue to rise until 2050. This underscores the persistent and substantial influence of behavioral risks on the burden of T2D.

The geographical and national disparities in ASMR and ASDR attributable to behavioral risks were pronounced. In 10 of the 21 regions, ASMR increased, with Eastern Europe, Central Asia and Southern Sub-Saharan Africa at the forefront. Several factors were posited to underpin this trend, although their individual contributions remain speculative. The aging global population and the concomitant rise in diabetes prevalence are associated with premature mortality and disability, amplifying the DALY burden. Industrialization and modernization have fostered sedentary behaviors and poor dietary habits, potentially exacerbating an increase in behavioral risks and a heavier burden of T2D. Additionally, advancements in medical diagnostics may partly account for the heightened ASDR observed [25, 26]. Oceania ranked highest in both ASMR and ASDR. Prior research indicates that the high diabetes prevalence, coupled with inadequate diabetes care, stems from complex structural influences [27]. Despite the highest diabetes prevalence, these Oceania countries often have the lowest treatment coverage, which is accompanied by disproportionately high ASDR and ASMR [28].

Our analysis identified four behavioral risks contributing to the ASMR and ASDR for T2D from 1990 to 2021: tobacco, dietary risks, high alcohol use and low physical activity. Modern lifestyle patterns—characterized by excessive intake of high-calorie, highly processed foods and insufficient physical activity—are closely associated with insulin resistance and dysregulated glucose homeostasis, which constitute the fundamental pathophysiological basis of T2D [29]. Dietary modifications can also alter the composition of the gut microbiota, thereby inducing low-grade systemic inflammation that further aggravates insulin resistance [30]. For individuals with T2D, improving dietary quality—such as increasing fiber intake and reducing the consumption of processed and sugar-rich foods—plays a critical role in both prevention and management of the disease [26]. Physical inactivity additionally heightens susceptibility to T2D by limiting skeletal muscle glucose uptake. Conversely, regular physical activity has been demonstrated to enhance insulin sensitivity and glucose metabolism both during and after exercise, thereby exerting protective effects on glucose homeostasis in individuals with T2D or at elevated risk [31]. Moreover, unhealthy behaviors such as smoking and excessive alcohol consumption have been well established as risk factors for T2D. Smoking, in particular, has been linked to increased T2D risk through mechanisms involving chronic low-grade inflammation, oxidative stress, and impaired insulin signaling. By contrast, the impact of alcohol consumption may vary across individuals, and further research is warranted to clarify these effects [32]. Studies have shown that alcohol consumption and the *rs56221195* genetic variant jointly influence insulin sensitivity and β-cell function [33]. In populations with high alcohol intake and metabolic vulnerability (e.g., elevated glucose or obesity), alcohol further exacerbates insulin resistance and impairs insulin secretion, while its impact on mortality and DALYs appears with a time lag [34]. In addition, GBD risk assessments are based on population-level exposure distributions. As global alcohol consumption rises—particularly in low- and middle-income countries—the overall population-level risk of T2D attributable to alcohol increases rapidly [35, 36]. Importantly, these findings highlight that adherence to a healthy lifestyle—including smoking cessation and moderate alcohol intake—can significantly reduce diabetes risk, lowering the incidence of latent autoimmune diabetes in adults (LADA) and T2D by 49% and 91%, respectively [37]. Collectively, these findings underscore that lifestyle factors shape T2D pathogenesis through intertwined metabolic, epigenetic, and inflammatory pathways, with dietary quality, physical activity, and weight management emerging as critical, modifiable targets for prevention and management.

The gender and age distribution of T2D deaths attributable to behavioral risks in 2021 revealed an association between advancing age and increased T2D mortality, peaking in women aged 80 to 84 and men aged 70 to 74. This aligns with previous studies [38–40], which may be explained by older patients' compromised physical health and multiple chronic comorbidities. The gender disparity in T2D burden is particularly striking. Analyses covering 1990–2021 and projections for the next 30 years both suggest that males consistently show higher mortality and DALY rates than females. This difference is largely attributed to gender-related behavioral and biological factors [41]. Smoking and high alcohol consumption, significant risk factors for T2D in males, partly contribute disproportionately to the T2D burden in this demographic.

The data also highlight a strong correlation between SDI values and the burden of T2D attributable to behavioral risks. High and high-middle SDI regions show lower ASMR and ASDR which may be related to improved socioeconomic conditions, as well as enhanced healthcare access and quality [42]. However, ASDR in high SDI regions increased rapidly from 1990 to 2021, and appeared to be associated with unhealthy diets, rising obesity, and global food system changes that promote energy-dense, nutrient-poor foods and limit physical activity [43, 44]. Aging populations in these regions further exacerbate the chronic disease burden [14]. Low-middle and low SDI regions had higher ASMRs than the global average. According to The Lancet Commission, in addition to underfunded and ill-prepared healthcare systems [3], low-income and middle-income countries were also beset by socioeconomic challenges such as poor nutrition, poverty, and physical inactivity [45].

This study represents a pioneering effort to quantify the global burden of T2D attributable to modifiable behavioral risks within the GBD framework. Despite the valuable insights provided by the GBD 2021 data, they share the limitations inherent to the GBD data. The accuracy of our estimates depends on the completeness and quality of country-level data, which may vary significantly [46]. To enhance robustness, GBD applies several methodological controls, including integration of multiple data sources, use of the Cause of Death Ensemble Model (CODEm) and DisMod-MR 2.1, as well as presentation of all estimates with 95% UIs [15]. To address data scarcity, we conduct analyses at the regional rather than country level, and cross-country comparisons should therefore be interpreted with caution. Additionally, for exposure measurement, this framework may omit relevant behavioral factors, and some data may be derived from sources with limited reliability, such as self-reports [47]. Furthermore, the GBD models quantify behavioral-attributable risk from available data, but these estimates are approximate and do not imply causal relationships [26]. Finally, the SDI employed in the GBD 2021 is a regional-level metric that does not consider potential patient-level confounders, limiting its applicability in individual risk assessment [16].

From a public health perspective, addressing the growing global burden of T2D requires comprehensive, evidence-based strategies and targeted public health interventions to promote healthy lifestyles and reduce exposure to modifiable behavioral risks. Strategies should include raising public awareness of the link between T2D and unhealthy dietary patterns, promoting balanced nutrition, and encouraging regular physical activity, alongside reshaping food environments through measures such as taxation of unhealthy foods and promotion of healthier options [48, 49]. Similarly, regulating alcohol availability, pricing, and marketing—while integrating behavioral counseling and culturally tailored public awareness campaigns—can help curb the escalating alcohol-related diabetes risk [50, 51]. Lessons from tobacco control, including advertising bans and targeted demographic interventions, further illustrate the effectiveness of such regulatory and educational approaches in mitigating behavioral risk factors [52]. Integrating these strategies into national health policies and resource allocation frameworks provides actionable pathways to translate epidemiological evidence into sustainable prevention efforts and mitigate future disease burden.

### Conclusion

The present study reveals a marked escalation in the global burden of T2D attributable to behavioral risks, with a particularly pronounced increase in ASDR. Males, older adults, and populations in low and low-middle SDI regions are disproportionately affected. Among the risk factors identified, dietary risks contribute the most, while high alcohol use exhibits the steepest increase. By identifying the most influential behavioral risks, our study provides evidence to guide future research and policy formulation, underscoring the need for targeted interventions as well as comprehensive T2D prevention and control strategies.
