# Supplementary material for: Global Burden of Type 2 Diabetes Attributable to Behavioral Risks: Insights and Projections to 2050 Based on the Global Burden of Disease Study 2021
Source: Int J Public Health. 2026 Mar 11;71:1608765. doi: 10.3389/ijph.2026.1608765 (PMC13014043; doi:10.3389/ijph.2026.1608765)
Supplement: Supplementary file 1 [file Supplementaryfile1.docx]

**Supplementary Material**

**Global burden of Type 2 Diabetes Attributable to Behavioral Risks: Insights and projections to 2050 based on the Global Burden of Disease Study 2021**

**Contents**

[Appendix 1. List of International Classification of Diseases (ICD) codes mapped to Type 2 Diabetes in GBD 2021 2](#_Toc30527)

[Appendix 2. GBD data collection, modeling/analysis, and dissemination 2](#_Toc18289)

[Appendix 3. Alcohol TMREL by region, age, and sex, 2020 2](#_Toc11515)

[Appendix 4. Risk factor estimation 3](#_Toc29928)

[Table S1: The Deaths number and DALYs of T2D attributable to behavioral risks in 1990 and 2021 at the global and regional level, and their AAPCs from 1990 to 2021 4](#_Toc691)

[Table S2 The global ASMR and ASDR of T2D attributable to four behavioral risks in 2021 at the Global and Regional Level 6](#_Toc31502)

[Table S3 The global deaths number and DALYs attributed to T2D associated with behavioral risks across all genders in different age groups in 2021 9](#_Toc30077)

[Table S4 The ASMR and ASDR attributed to T2D associated with behavioral risk factors across all genders in different age groups in 2021 10](#_Toc16796)

[Table S5 The ASMR, ASDR, Deaths number, and DALYs attributed to T2D associated with four behavioral risk factors by gender in 2021 11](#_Toc14629)

[Figure S1. The Deaths numbers and DALYs of T2D attributable to behavioral risks in 1990 and 2021 at the global and regional level, and their AAPCs from 1990 to 2021. 12](#_Toc21575)

[Figure S2. The ASMR and ASDR of T2D attributed to the secondary classification of four behavioral risks in 2021. 13](#_Toc32095)

[Figure S3. Trends in ASMR and ASDR of T2D attributable to behavioral risks globally and across SDI regions from 1990 to 2021.. 14](#_Toc21396)

[Figure S4. Trends in ASMR and ASDR of T2D attributable to behavioral risks globally and across SDI regions from 1990 to 2021. 15](#_Toc22957)

[Figure S5. The Death numbers and DALYs of T2D attributable to behavioral risks, globally and for 21 GBD regions, by SDI from 1990 to 2021.. 16](#_Toc30123)

[Figure S6. The ASMR and ASDR of T2D attributable to behavioral risks, globally and for 21 GBD regions, by SDI from 1990 to 2021. . 17](#_Toc32131)

[Figure S7. The relationship between AAPCs in ASMR and ASDR of T2D attributable to behavioral risks with SDI in 2021. 18](#_Toc24179)

[Figure S8. Temporal trends of the number and age-standardized rates for T2D attributable to behavioral risks at the global level and by sex from 1990 to 2050. 19](#_Toc28263)

Appendix 1. List of International Classification of Diseases (ICD) codes mapped to Type 2 Diabetes in GBD 2021

| Cause | ICD9 | ICD10 |
| --- | --- | --- |
| Type 2 Diabetes | 250 | E11-E11.1 and E11.3-E11.9 |

Appendix 2. GBD data collection, modeling/analysis, and dissemination

The Global Burden of Disease (GBD) study is a comprehensive research aimed at systematically assessing the health status and disease burden of populations worldwide. An international network comprising over 11,500 collaborators from 164 countries and territories contributed to the generation of GBD metrics through data provision, review, and analysis. GBD data collection involves diverse sources, including epidemiological surveys, vital registration systems, disease surveillance systems, hospital records, and additional sources such as academic papers and policy reports (https://ghdx. healthdata.org/gbd-2021/sources). The data is standardized using the International Classification of Diseases (ICD) codes to ensure accuracy and comparability (https://ghdx.healthdata.org/record/ ihme-data/gbd-2021-cause-icd-code-mappings). Sophisticated modeling tools, such as DisMod-MR and Spatiotemporal Gaussian Process Regression (ST-GPR), are employed to estimate prevalence, incidence, and mortality rates. Data processing includes corrections for heterogeneity and biases, as well as uncertainty analysis through Monte Carlo simulations. Key health metrics include Years of Life Lost (YLLs), Years Lived with Disability (YLDs), and Disability-Adjusted Life Years (DALYs) (https://www.healthdata.org/gbd/methods-appendices-2021/cancers). Dissemination of GBD findings is achieved through scientific publications (https://www.healthdata.org/research-analysis/gbd- publications), and interactive tools like GBD Compare and Viz Hub (https://www.healthdata.org/ research-analysis/gbd-data). These tools facilitate the exploration and comparison of health data across regions and time periods. The primary goal of GBD findings is to provide a comprehensive framework for understanding global and local health trends, thereby supporting evidence-based health decision-making and resource allocation.

**Appendix 3. Alcohol TMREL by region, age, and sex, 2020**


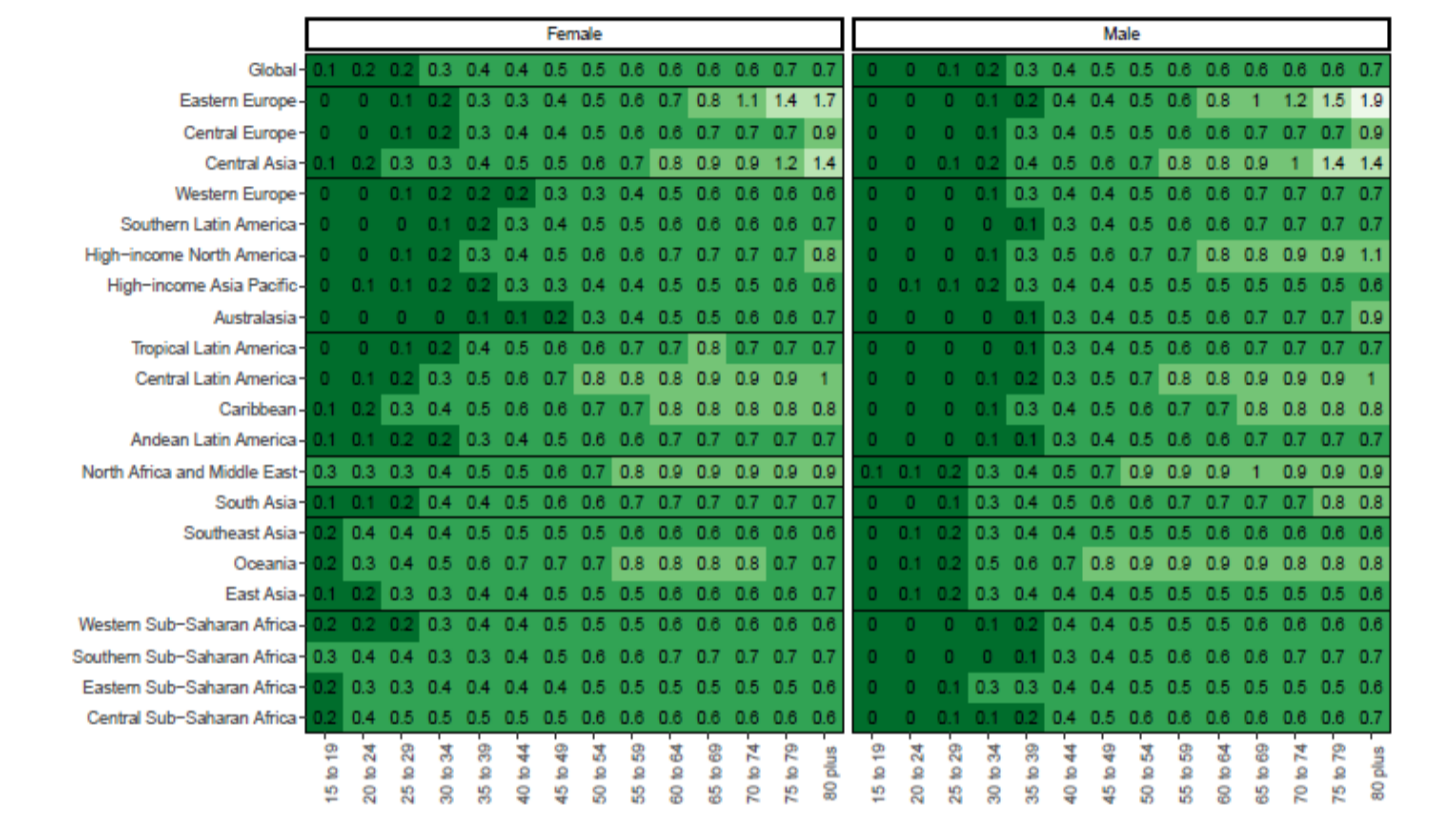


**Appendix 4. Risk factor estimation**

GBD applies a comparative risk assessment approach: (1) defining risk–outcome pairs based on systematic evidence; (2) estimating exposure distributions from surveys, surveillance, and published data using statistical models such as ST-GPR; (3) specifying the theoretical minimum risk exposure level (TMREL) as the counterfactual; (4) calculating population attributable fractions (PAFs) by integrating exposure distributions with relative risks; (5) deriving summary exposure values (SEVs) to enable cross-population comparisons; (6) applying mediation adjustments to avoid double counting where risks act through intermediate pathways; and (7) estimating attributable deaths and DALYs by multiplying cause-specific burden by PAFs, stratified by age, sex, region, and SDI.

Table S1: The Deaths number and DALYs of T2D attributable to behavioral risks in 1990 and 2021 at the global and regional level, and their AAPCs from 1990 to 2021 (Global Burden of Diseases Study, 21 Global Burden of Diseases regions, 1990–2021).

|  | **Number of cases (95% UI)** | | | | **Average Annual Percentage Changes**  **(AAPCs) from 1990 to 2021 (95% CI)** | |
| --- | --- | --- | --- | --- | --- | --- |
|  | **1990** | | **2021** | |  |  |
|  | **Deaths number*10^3^** | **DALYs number*10^3^** | **Deaths number*10^3^** | **DALYs number*10^3^** | **Deaths number** | **DALYs number** |
| **Global** | 267.95 (159.11,347.93) | 10493.59 (5947.43,14119.7) | 626.66 (368.98,822.57) | 30187.75 (16700.76,42159.85) | 2.75 (2.58,2.92)^***^ | 3.47 (3.40,3.55)^***^ |
| **SDI category** |  |  |  |  |  |  |
| High SDI | 70.89 (41.66,92.15) | 2602.83 (1496.03,3490.86) | 99.83 (55.53,131.90) | 6298.02 (3351.94,9118.07) | 1.11 (0.97,1.26)^***^ | 2.91 (2.84,2.97)^***^ |
| High-middle SDI | 54.06 (32.74,69.07) | 2287.26 (1318.76,3058.98) | 108.84 (64.82,143.42) | 5710.45 (3235.41,7954.57) | 2.32 (2.03,2.61)^***^ | 3.04 (2.93,3.16)^***^ |
| Middle SDI | 68.38 (45.22,87.21) | 2913.27 (1816.20,3894.65) | 208.56 (129.51,271.67) | 9613.72 (5708.1,13312.69) | 3.72 (3.50,3.94)^***^ | 3.94 (3.86,4.03)^***^ |
| Low-middle SDI | 49.94 (29.19,66.72) | 1836.60 (1051.25,2482.53) | 155.73 (91.16,204.69) | 6336.12 (3616.23,8784.35) | 3.75 (3.52,3.99)^***^ | 4.09 (3.99,4.18)^***^ |
| Low SDI | 24.24 (10.53,34.97) | 836.55 (359.24,1213.32) | 52.92 (24.39,75.43) | 2193.67 (945.21,3192.92) | 2.57 (2.47,2.66)^***^ | 3.16 (3.11,3.22)^***^ |
| **Southeast Asia, East Asia, and Oceania Region** | 52.15 (35.15,67.11) | 2476.29 (1559.33,3299.9) | 142.23 (93.79,189.07) | 7727.19 (4708.64,10785.73) | 3.32 (3.22,3.42)^***^ | 3.78 (3.64,3.93)^***^ |
| East Asia | 29.23 (19.42,38.51) | 1652.15 (1017.48,2281.39) | 78.45 (50.17,109.64) | 5118.18 (2963.8,7333.47) | 3.28 (3.11,3.46)^***^ | 3.76 (3.59,3.92)^***^ |
| Southeast Asia | 21.81 (14.53,28.30) | 784.61 (498.22,1025.90) | 60.84 (41.53,78.92) | 2486.97 (1634.33,3289.41) | 3.37 (3.29,3.44)^***^ | 3.78 (3.69,3.87)^***^ |
| Oceania | 1.11 (0.62,1.60) | 39.53 (21.97,56.11) | 2.94 (1.66,4.05) | 122.04 (68.20,167.64) | 3.22 (3.15,3.3)^***^ | 3.69 (3.64,3.74)^***^ |
| **Central Europe, Eastern Europe, and**  **Central Asia Region** | 16.99 (9.22,22.26) | 920.93 (483.15,1258.95) | 43.80 (23.46,58.04) | 2113.24 (1095.35,2943.17) | 3.14 (2.34,3.94)^***^ | 2.72 (2.39,3.04)^***^ |
| Central Asia | 2.12 (1.03,2.82) | 105.14 (51.46,146.82) | 6.71(3.22,9.33) | 385.99 (182.94,543.93) | 3.75 (3.39,4.11)^***^ | 4.28 (4.03,4.53)^***^ |
| Central Europe | 9.23 (5.45,11.87) | 435.76 (251.13,586.07) | 15.76 (9.28,20.81) | 775.48 (437.52,1078.64) | 1.85 (1.42,2.28)^***^ | 1.94 (1.72,2.17)^***^ |
| Eastern Europe | 5.63 (2.86,7.48) | 380.02 (183.36,537.05) | 21.32 (11.16,28.22) | 951.77 (481.19,1323.68) | 4.42 (3.71,5.14)^***^ | 3.02 (2.77,3.26)^***^ |
| **High-income Region** | 85.49 (49.93,110.82) | 2929.38 (1675.17,3915) | 105.86 (58.31,139.60) | 6341.55 (3332.28,9206.34) | 0.72 (0.50,0.93)^***^ | 2.53 (2.48,2.58)^***^ |
| High-income Asia Pacific | 7.74 (5.08,9.81) | 441.65 (275.74,592.59) | 8.47 (4.98,11.36) | 998.76 (546.10,1503.23) | 0.06 (-0.65,0.77) | 2.59 (2.38,2.80)^***^ |
| Australasia | 1.35 (0.76,1.78) | 46.25 (25.52,62.78) | 2.55 (1.38,3.45) | 114.71 (57.98,166.55) | 2.13 (1.42,2.85)^***^ | 2.95 (2.55,3.35)^***^ |
| Western Europe | 45.08 (26.77,58.4) | 1305.08 (754.78,1731.34) | 47.86 (26.28,63.74) | 2107.78 (1126.29,3000.91) | 0.25 (0.06,0.43)^***^ | 1.57 (1.48,1.65)^***^ |
| Southern Latin America | 5.08 (2.68,6.73) | 166.43 (87.58,225.7) | 6.82 (3.42,9.12) | 338.29 (166.79,494.73) | 0.84 (0.37,1.32)^***^ | 2.41 (2.08,2.73)^***^ |
| High-income North America | 26.23 (14.44,34.28) | 969.96 (534.81,1319.17) | 40.17 (21.54,53.06) | 2782.01 (1450.41,4013.73) | 1.41 (1.18,1.65)^***^ | 3.50 (3.34,3.66)^***^ |
| **Latin America and Caribbean Region** | 29.63 (18.33,38.3) | 1133.68 (665.46,1533.09) | 76.39 (42.99,105.76) | 3186.20 (1665.51,4579.29) | 3.11 (2.73,3.49)^***^ | 3.42 (3.19,3.66)^***^ |
| Caribbean | 4.08 (2.34,5.47) | 141.36 (78.34,194.07) | 6.87 (3.81,9.66) | 312.41 (169.61,437.92) | 1.73 (1.43,2.03)^***^ | 2.62 (2.47,2.77)^***^ |
| Andean Latin America | 1.19 (0.63,1.68) | 43.13 (21.44,61.85) | 4.10 (2.13,6.00) | 174.03 (86.76,259.12) | 4.16 (3.84,4.49)^***^ | 4.64 (4.27,5.02)^***^ |
| Central Latin America | 13.09 (8.22,17.07) | 510.80 (295.06,697.45) | 37.81 (20.53,53.24) | 1556.52 (812.27,2249.40) | 3.56 (2.63,4.50)^***^ | 3.69 (3.24,4.15)^***^ |
| Tropical Latin America | 11.27 (7.39,14.38) | 438.39 (274.01,573.41) | 27.62 (15.92,36.85) | 1143.24 (607.65,1625.41) | 2.96 (2.51,3.4)^***^ | 3.17 (3.12,3.21)^***^ |
| **North Africa and Middle East Region** | 14.35 (9.39,18.68) | 564.41 (346.26,757.78) | 46.91 (29.31,61.30) | 2665.99 (1582.33,3678.34) | 3.92 (3.79,4.05)^***^ | 5.13 (5.08,5.18)^***^ |
| **South Asia Region** | 42.83 (25.93,57.21) | 1617.47 (939.28,2191.21) | 148.43 (86.79,199.96) | 5846.15 (3267.94,8208.25) | 4.21 (3.99,4.42)^***^ | 4.23 (4.07,4.38)^***^ |
| **Sub-Saharan Africa Region** | 26.51 (11.51,38.02) | 851.43 (362.63,1232.80) | 63.04 (29.47,88.50) | 2307.43 (1027.24,3323.83) | 2.87 (2.82,2.92)^***^ | 3.28 (3.24,3.33)^***^ |
| Central Sub-Saharan Africa | 3.39 (1.23,5.29) | 113.64 (38.07,173.46) | 8.10 (2.70,12.63) | 328.26 (104.58,502.92) | 2.87 (2.73,3.01)^***^ | 3.49 (3.42,3.55)^***^ |
| Eastern Sub-Saharan Africa | 10.63 (3.71,15.90) | 333.18 (114.42,495.62) | 18.12 (7.21,26.93) | 638.24 (241.39,943.41) | 1.74 (1.66,1.82)^***^ | 2.11 (2.03,2.18)^***^ |
| Southern Sub-Saharan Africa | 4.59 (2.77,6.03) | 143.35 (84.73,190.24) | 15.93 (8.81,21.14) | 507.26 (277.44,683.57) | 4.22 (3.61,4.84)^***^ | 4.23 (3.73,4.73)^***^ |
| Western Sub-Saharan Africa | 7.90 (3.71,11.26) | 261.26 (119.09,373.37) | 20.89 (10.08,29.4) | 833.67 (379.09,1223.38) | 3.20 (3.12,3.27)^***^ | 3.81 (3.75,3.87)^***^ |

Abbreviations: T2D, Type 2 Diabetes; DALYs, Disability-adjusted life years; SDI, Socio Demographic Index; UI, Uncertainty Interval; AAPCs, Average Annual Percentage Changes; CI, Confidence Interval.

* P < 0.05,** P < 0.01,*** P < 0.001.

Table S2 The global ASMR and ASDR of T2D attributable to four behavioral risks in 2021 at the Global and Regional Level (Global Burden of Diseases Study, 21 Global Burden of Diseases regions, 1990–2021).

| **Region** | **Age-Standardized Rate Per 100,000 People (95% UI)** | | | | | | | |
| --- | --- | --- | --- | --- | --- | --- | --- | --- |
|  | **Tobacco** | | **High alcohol use** | | **Dietary risks** | | **Low physical activity** | |
|  | **Death** | **DALYs** | **Death** | **DALYs** | **Death** | **DALYs** | **Death** | **DALYs** |
| Global | 1.91 (1.20,2.62) | 102.08 (64.15,146.20) | 0.34 (0.12,0.64) | 15.43 (3.67,32.25) | 4.52 (0.88,7.36) | 221.34 (47.97,368.92) | 1.80 (0.79,2.75) | 64.27 (28.01,100.49) |
| High SDI | 0.88 (0.61,1.19) | 82.49 (54.62,118.05) | 0.34 (0.12,0.65) | 22.03 (2.31,50.62) | 3.16 (0.72,4.94) | 247.38 (62.69,408.23) | 0.88 (0.37,1.37) | 52.10 (23.03,84.44) |
| High-middle SDI | 1.43 (0.90,1.94) | 96.68 (60.19,140.23) | 0.26 (0.10,0.47) | 13.16 (2.58,28.12) | 3.57 (0.74,5.74) | 192.42 (41.84,324.99) | 1.30 (0.58,1.98) | 48.14 (21.04,75.47) |
| Middle SDI | 2.32 (1.43,3.21) | 111.44 (68.02,158.48) | 0.38 (0.14,0.70) | 15.50 (4.88,31.45) | 4.43 (0.78,7.49) | 197.22 (39.52,337.88) | 2.32 (1.03,3.53) | 75.81 (33.18,117.39) |
| Low-middle SDI | 3.45 (2.07,4.83) | 131.70 (80.09,187.90) | 0.33 (0.11,0.67) | 11.58 (2.50,25.65) | 7.01 (1.34,11.53) | 254.14 (55.93,422.80) | 3.22 (1.39,4.96) | 88.59 (38.29,137.73) |
| Low SDI | 2.36 (1.37,3.40) | 85.67 (52.06,123.08) | 0.29 (0.03,0.67) | 7.99 (-1.52,23.03) | 8.68 (1.33,14.53) | 292.19 (50.31,492.72) | 2.33 (0.96,3.70) | 62.74 (26.40,99.23) |
| **Southeast Asia, East Asia, and  Oceania Region** | 2.04 (1.27,2.76) | 112.98 (69.73,162.68) | 0.20 (0.08,0.38) | 10.39 (2.62,21.34) | 2.45 (0.32,4.37) | 137.98 (20.02,249.08) | 1.39 (0.62,2.16) | 50.63 (22.10,77.98) |
| East Asia | 1.42 (0.88,2.00) | 99.82 (61.29,146.50) | 0.15 (0.06,0.29) | 9.24 (2.06,20.52) | 1.93 (0.22,3.50) | 134.85 (17.33,247.24) | 0.94 (0.42,1.51) | 37.81 (16.28,60.02) |
| Southeast Asia | 4.01 (2.44,5.53) | 156.21 (98.55,216.72) | 0.37 (0.14,0.72) | 13.99 (4.85,27.73) | 3.96 (0.64,6.91) | 149.62 (26.93,267.51) | 2.99 (1.32,4.49) | 94.14 (40.25,145.48) |
| Oceania | 13.52 (7.63,19.49) | 487.53 (290.44,695.67) | 0.75 (0.23,1.45) | 26.47 (6.40,53.27) | 26.27 (4.25,44.14) | 862.05 (154.31,1465.63) | 8.68 (3.61,13.94) | 268.48 (110.53,431.69) |
| **Central Europe, Eastern Europe, and Central Asia Region** | 1.35 (0.86,1.84) | 86.62 (56.01,122.51) | 0.36 (0.15,0.66) | 18.87 (4.17,39.68) | 4.87 (1.10,7.53) | 248.15 (61.29,399.13) | 1.17 (0.50,1.79) | 41.67 (17.61,65.92) |
| Central Asia | 1.85 (1.10,2.65) | 103.62 (61.62,150.72) | 0.32 (0.11,0.60) | 16.47 (3.94,32.85) | 6.54 (1.52,10.24) | 343.63 (89.24,548.12) | 0.91 (0.39,1.45) | 37.44 (16.05,60.33) |
| Central Europe | 1.56 (1.01,2.14) | 105.36 (68.85,151.40) | 0.54 (0.20,1.02) | 28.31 (6.31,60.22) | 4.64 (1.05,7.24) | 260.14 (64.84,428.55) | 1.34 (0.57,2.04) | 53.07 (23.28,85.99) |
| Eastern Europe | 1.06 (0.68,1.45) | 70.17 (46.07,98.49) | 0.24 (0.09,0.46) | 13.13 (1.61,28.68) | 4.47 (1.01,6.90) | 209.93 (50.23,333.66) | 1.08 (0.44,1.70) | 34.64 (14.43,54.83) |
| **High-income Region** | 0.78 (0.53,1.05) | 75.89 (50.45,108.59) | 0.35 (0.12,0.68) | 21.64 (0.96,51.46) | 3.23 (0.77,5.01) | 247.91 (64.63,404.91) | 0.84 (0.35,1.29) | 48.62 (21.14,79.04) |
| High-income Asia Pacific | 0.42 (0.29,0.57) | 76.69 (48.08,115.25) | 0.09 (0.02,0.21) | 10.58 (-6.22,37.54) | 0.99 (0.20,1.61) | 186.59 (43.21,321.83) | 0.40 (0.17,0.64) | 55.05 (23.10,90.40) |
| Australasia | 0.54 (0.34,0.75) | 39.96 (25.91,57.46) | 0.38 (0.09,0.76) | 15.65 (-1.34,40.33) | 3.15 (0.61,4.94) | 169.44 (35.79,286.11) | 1.09 (0.46,1.70) | 47.32 (20.97,76.47) |
| Western Europe | 0.65 (0.44,0.88) | 58.42 (38.23,84.07) | 0.36 (0.11,0.68) | 15.38 (-2.41,42.56) | 3.12 (0.70,4.83) | 195.78 (50.12,317.27) | 0.94 (0.40,1.45) | 41.73 (18.57,66.22) |
| Southern Latin America | 1.49 (0.95,2.08) | 95.62 (60.95,135.12) | 0.46 (0.12,0.97) | 20.55 (0.80,50.04) | 6.04 (1.47,9.22) | 314.16 (83.59,514.34) | 0.91 (0.36,1.47) | 36.76 (15.48,59.81) |
| High-income North America | 1.10 (0.77,1.46) | 98.61 (66.32,140.28) | 0.50 (0.17,0.91) | 37.04 (7.55,74.61) | 4.54 (1.15,6.96) | 354.01 (96.56,574.67) | 0.99 (0.40,1.56) | 57.19 (24.10,92.69) |
| **Latin America and Caribbean Region** | 2.58 (1.63,3.53) | 113.74 (72.61,159.81) | 0.78 (0.25,1.44) | 29.76 (5.61,61.78) | 7.68 (1.41,13.00) | 324.79 (65.87,559.91) | 3.20 (1.37,4.97) | 104.59 (44.26,164.15) |
| Caribbean | 2.48 (1.54,3.47) | 134.97 (84.73,191.26) | 0.67 (0.21,1.26) | 32.12 (6.61,67.83) | 7.60 (1.32,13.04) | 358.72 (72.30,623.26) | 3.63 (1.57,5.73) | 135.27 (58.91,219.06) |
| Andean Latin America | 1.26 (0.79,1.81) | 57.41 (36.16,82.11) | 0.40 (0.03,0.89) | 14.55 (-2.49,37.57) | 4.51 (0.82,7.91) | 192.80 (41.00,334.98) | 1.65 (0.69,2.64) | 53.89 (23.08,86.04) |
| Central Latin America | 3.00 (1.87,4.11) | 125.99 (78.85,175.32) | 1.17 (0.38,2.15) | 42.99 (8.48,84.04) | 9.68 (2.09,16.74) | 399.29 (90.88,692.80) | 3.28 (1.43,5.09) | 106.24 (45.54,169.23) |
| Tropical Latin America | 2.48 (1.56,3.47) | 110.13 (70.91,156.82) | 0.51 (0.15,0.99) | 19.80 (3.55,43.53) | 6.47 (0.90,11.09) | 274.73 (42.57,474.23) | 3.39 (1.40,5.23) | 108.19 (44.79,168.94) |
| **North Africa and Middle East Region** | 3.35 (1.96,4.78) | 168.99 (98.42,244.61) | 0.07 (0.03,0.12) | 3.73 (1.23,7.14) | 6.92 (1.78,11.04) | 329.22 (95.87,548.70) | 3.56 (1.54,5.50) | 139.58 (60.62,219.11) |
| **South Asia Region** | 3.09 (1.80,4.48) | 112.34 (65.84,162.07) | 0.32 (0.10,0.61) | 11.75 (2.58,25.95) | 6.71 (1.42,11.08) | 234.58 (55.00,390.49) | 3.27 (1.41,5.05) | 82.00 (37.08,128.77) |
| **Sub-Saharan Africa Region** | 2.48 (1.47,3.47) | 81.32 (49.41,114.94) | 0.70 (0.17,1.51) | 17.82 (1.56,42.96) | 10.84 (1.71,17.9) | 325.69 (55.95,551.79) | 3.46 (1.44,5.38) | 84.42 (35.23,131.84) |
| Central Sub-Saharan Africa | 2.42 (1.45,3.60) | 89.27 (54.83,128.88) | 0.40 (-0.17,1.33) | 8.00 (-13.67,41.84) | 13.03 (0.69,23.34) | 409.39 (26.58,716.04) | 3.88 (1.64,6.46) | 94.06 (40.10,157.87) |
| Eastern Sub-Saharan Africa | 2.18 (1.36,3.07) | 67.58 (43.04,94.73) | 0.36 (-0.06,1.01) | 8.18 (-5.20,30.32) | 9.52 (1.22,16.22) | 268.20 (38.50,457.43) | 1.67 (0.66,2.75) | 39.73 (15.73,64.62) |
| Southern Sub-Saharan Africa | 6.29 (3.63,8.95) | 196.51 (116.91,276.18) | 1.73 (0.62,3.29) | 47.59 (14.51,94.26) | 18.17 (2.82,30.34) | 523.89 (83.02,886.11) | 9.89 (4.18,15.08) | 239.25 (102.53,361.94) |
| Western Sub-Saharan Africa | 1.56 (0.85,2.30) | 56.26 (31.99,83.30) | 0.73 (0.25,1.51) | 19.54 (4.29,43.26) | 9.05 (1.98,14.73) | 290.63 (65.71,476.82) | 2.77 (1.12,4.36) | 71.51 (29.26,112.18) |

Abbreviations: T2D, Type 2 Diabetes; ASMR, Age-­standardized mortality rate; ASDR, Age-­standardized disability-­adjusted life years rate; DALYs, Disability-adjusted life years; UI, Uncertainty Interval; SDI, Socio Demographic Index.

Table S3 The global deaths number and DALYs attributed to T2D associated with behavioral risks across all genders in different age groups in 2021 (Global Burden of Diseases Study, 2021).

| **AGE** | **Number of cases (95% UI)** | | | |
| --- | --- | --- | --- | --- |
|  | **Deaths Number** | | **DALYs Number** | |
|  | **male** | **female** | **male** | **female** |
| **All ages** | 314069.09 (193484.25,409868.51) | 312591.49 (172338.46,418380.27) | 16121012.76 (9433079.59,22249325.01) | 14066735.04 (7395370.17,20116752.67) |
| **15-19 years** | -22.21 (-35.22,-8.99) | -10.41 (-18.12,-3.26) | -3997.26 (-6645.71,-1738.47) | -1694.77 (-2989.51,-591.82) |
| **20-24 years** | -34.78 (-59.08,-7.42) | -15.88 (-30.35,-2.78) | -10208.92 (-18542.17,-1114.69) | -4189.55 (-7684.86,-865.78) |
| **25-29 years** | 483.83 (143.27,754.50) | 631.88 (228.84,939.20) | 192916.32 (61434.44,323914.5) | 200578.71 (81961.41,324243.51) |
| **30-34 years** | 1673.51 (816.31,2289.34) | 1049.58 (437.07,1506.13) | 423004.13 (213155.85,630582.95) | 313673.08 (142907.27,491791.42) |
| **35-39 years** | 2449.20 (1304.28,3339.96) | 1743.41 (768.01,2502.13) | 571763.99 (304380.93,845125.74) | 423704.41 (195021.3,653105.32) |
| **40-44 years** | 5040.20 (2809.88,6797.42) | 3341.96 (1519.03,4695.98) | 821754.31 (447715.28,1194718.37) | 584810.57 (271422.07,885285.55) |
| **45-49 years** | 11064.27 (6461.11,14522.66) | 7378.92 (3552.56,10288.87) | 1272956.82 (729662.85,1790307.67) | 901341.23 (434402.64,1331037.75) |
| **50-54 years** | 19042.46 (11481.92,25086.34) | 13209.94 (6663.58,18204.07) | 1757674.35 (1040266.76,2440026.8) | 1273360.77 (628565.39,1832538.45) |
| **55-59 years** | 28029.41 (17185.82,36373.97) | 20672.49 (10812.27,28033.13) | 2115325.1 (1263579.48,2882840.87) | 1632673.49 (842103.25,2350032.96) |
| **60-64 years** | 37053.98 (22651.22,48170.76) | 30750.54 (16521.95,41328.12) | 2246790.04 (1367557.77,3103059.72) | 1891221.62 (1005090.69,2669230.7) |
| **65-69 years** | 44392.82 (27457.12,57321.49) | 39836.39 (21262.96,53866.81) | 2228418.9 (1343434.84,3062407.1) | 1989361.73 (1062142.64,2837112.14) |
| **70-74 years** | 47048.88 (29263.06,61904.73) | 43774.90 (24482.36,59095.48) | 1872963.35 (1102416.7,2603864.37) | 1737309.06 (953935.05,2468084.48) |
| **75-79 years** | 42402.07 (25978.29,55818.68) | 44208.48 (24633.84,59201.15) | 1261153.26 (750376.09,1726568.15) | 1287169.41 (703811.83,1802203.65) |
| **80-84 years** | 34807.00 (20953.04,46101.76) | 44960.63 (25605.68,60751.12) | 775254.96 (465310.54,1064544.87) | 960117.5 (550866.94,1331840.43) |
| **85-89 years** | 25499.64 (15105.02,33589.89) | 33400.82 (18880.17,45725.18) | 405521.17 (239524.35,555502.59) | 538767.89 (306359.09,750748.14) |
| **90-94 years** | 11846.48 (6833.19,15753.86) | 19497.92 (10682.04,26633.89) | 151326.41 (88181.39,203899.98) | 249094.44 (139279.59,341394.64) |
| **95+ years** | 3292.35 (1804.72,4488.50) | 8159.90 (4215.91,11348.62) | 38395.85 (21682.79,52152.53) | 89435.47 (48258.74,123238.69) |

Abbreviations: T2D, Type 2 Diabetes; DALYs, Disability-adjusted life years; UI, Uncertainty Interval.

Table S4 The ASMR and ASDR attributed to T2D associated with behavioral risk factors across all genders in different age groups in 2021 (Global Burden of Diseases Study, 2021).

| **AGE** | **Age-Standardized Rate Per 100,000 People (95% UI)** | | | |
| --- | --- | --- | --- | --- |
|  | **Deaths Rate** | | **DALYs Rate** | |
|  | **male** | **female** | **male** | **female** |
| **Age-standardized** | 8.27 (5.04,10.81) | 6.72 (3.70,8.99) | 391.31 (228.68,539.76) | 309.16 (161.81,442.68) |
| **15-19 years** | -0.01 (-0.01,0.00) | 0.00 (-0.01,0.00) | -1.25 (-2.07,-0.54) | -0.56 (-0.98,-0.19) |
| **20-24 years** | -0.01 (-0.02,0.00) | -0.01 (-0.01,0.00) | -3.36 (-6.11,-0.37) | -1.43 (-2.62,-0.29) |
| **25-29 years** | 0.16 (0.05,0.25) | 0.22 (0.08,0.32) | 64.88 (20.66,108.93) | 68.93 (28.17,111.43) |
| **30-34 years** | 0.55 (0.27,0.75) | 0.35 (0.15,0.50) | 138.44 (69.76,206.38) | 104.93 (47.81,164.52) |
| **35-39 years** | 0.87 (0.46,1.18) | 0.63 (0.28,0.90) | 201.99 (107.53,298.56) | 152.52 (70.20,235.10) |
| **40-44 years** | 2.00 (1.11,2.70) | 1.35 (0.61,1.89) | 325.88 (177.55,473.79) | 235.73 (109.40,356.84) |
| **45-49 years** | 4.65 (2.72,6.11) | 3.13 (1.51,4.37) | 535.17 (306.76,752.67) | 382.50 (184.35,564.85) |
| **50-54 years** | 8.58 (5.17,11.30) | 5.93 (2.99,8.17) | 791.82 (468.63,1099.21) | 571.16 (281.94,821.98) |
| **55-59 years** | 14.39 (8.83,18.68) | 10.28 (5.38,13.95) | 1086.32 (648.91,1480.47) | 812.26 (418.95,1169.15) |
| **60-64 years** | 23.82 (14.56,30.97) | 18.69 (10.04,25.12) | 1444.54 (879.25,1995.06) | 1149.60 (610.96,1622.52) |
| **65-69 years** | 33.67 (20.83,43.48) | 27.66 (14.77,37.41) | 1690.33 (1019.04,2322.94) | 1381.42 (737.55,1970.09) |
| **70-74 years** | 48.81 (30.36,64.22) | 40.00 (22.37,53.99) | 1943.08 (1143.69,2701.34) | 1587.34 (871.59,2255.03) |
| **75-79 years** | 70.92 (43.45,93.36) | 61.32 (34.17,82.11) | 2109.42 (1255.09,2887.88) | 1785.31 (976.19,2499.67) |
| **80-84 years** | 94.97 (57.17,125.78) | 88.28 (50.28,119.28) | 2115.19 (1269.54,2904.48) | 1885.13 (1081.59,2614.98) |
| **85-89 years** | 147.80 (87.55,194.69) | 117.32 (66.32,160.61) | 2350.48 (1388.33,3219.81) | 1892.46 (1076.11,2637.06) |
| **90-94 years** | 203.25 (117.24,270.29) | 161.66 (88.57,220.83) | 2596.32 (1512.93,3498.33) | 2065.31 (1154.81,2830.60) |
| **95+ years** | 217.74 (119.36,296.85) | 207.20 (107.05,288.16) | 2539.34 (1434.01,3449.15) | 2270.93 (1225.38,3129.26) |

Abbreviations: T2D, Type 2 Diabetes; DALYs, Disability-adjusted life years; UI, Uncertainty Interval.

Table S5 The ASMR, ASDR, Deaths number, and DALYs attributed to T2D associated with four behavioral risk factors by gender in 2021 (Global Burden of Diseases Study, 2021).

|  | Gender | **Tobacco** | **Dietary risks** | **Alcohol use** | **Low physical activity** |
| --- | --- | --- | --- | --- | --- |
| **ASMR per 100,000**  **(95% UI)** | Both | 1.91 (1.20,2.62) | 4.52 (0.88,7.36) | 0.34 (0.12,0.64) | 1.80 (0.79,2.75) |
|  | Male | 2.66 (1.88,3.47) | 4.85 (0.92,7.95) | 0.64 (0.24,1.20) | 1.50 (0.65,2.34) |
|  | Female | 1.27 (0.63,1.94) | 4.25 (0.85,6.98) | 0.09 (0.02,0.18) | 2.03 (0.90,3.11) |
| **Deaths Number*10**3  **(95% UI)** | Both | 163.22 (103.03,223.44) | 381.42 (74.33,620.91) | 28.63 (10.09,54.91) | 149.21 (65.19,228.32) |
|  | Male | 104.35 (74.07,135.52) | 183.72 (34.69,301.29) | 24.62 (9.11,46.55) | 54.11 (23.39,84.09) |
|  | Female | 58.87 (29.06,89.84) | 197.7 (39.64,324.53) | 4.01 (1.00,8.34) | 95.11 (42.06,145.45) |
| **ASDR per 100,000**  **(95% UI)** | Both | 102.08 (64.15,146.20) | 221.34 (47.97,368.92) | 15.43 (3.67,32.25) | 64.27 (28.01,100.49) |
|  | Male | 140.51 (97.69,190.99) | 236.94 (50.42,397.97) | 28.73 (8.75,57.51) | 52.92 (22.89,83.10) |
|  | Female | 66.58 (32.89,103.49) | 207.18 (45.75,344.06) | 3.49 (-0.46,9.41) | 74.38 (32.27,115.78) |
| **DALYs Number*10**3  **(95% UI)** | Both | 8898.6 (5598.93,12721.29) | 19146.81 (4147.24,31937.62) | 1359.61 (338.15,2834.92) | 5523.05 (2407.13,8638.53) |
|  | Male | 5882.15 (4096.41,8000.50) | 9750.52 (2078.05,16367.49) | 1192.97 (350.08,2394.48) | 2094.65 (907.07,3290.69) |
|  | Female | 3016.45 (1493.61,4680.82) | 9396.29 (2069.19,15612.53) | 166.63 (-13.12,439.49) | 3428.4 (1487.01,5330.56) |

Abbreviations: T2D, Type 2 Diabetes; ASMR, Age-­standardized mortality rate; ASDR, Age-­standardized disability-­adjusted life years rate; DALYs, Disability-adjusted life years; UI, Uncertainty Interval.


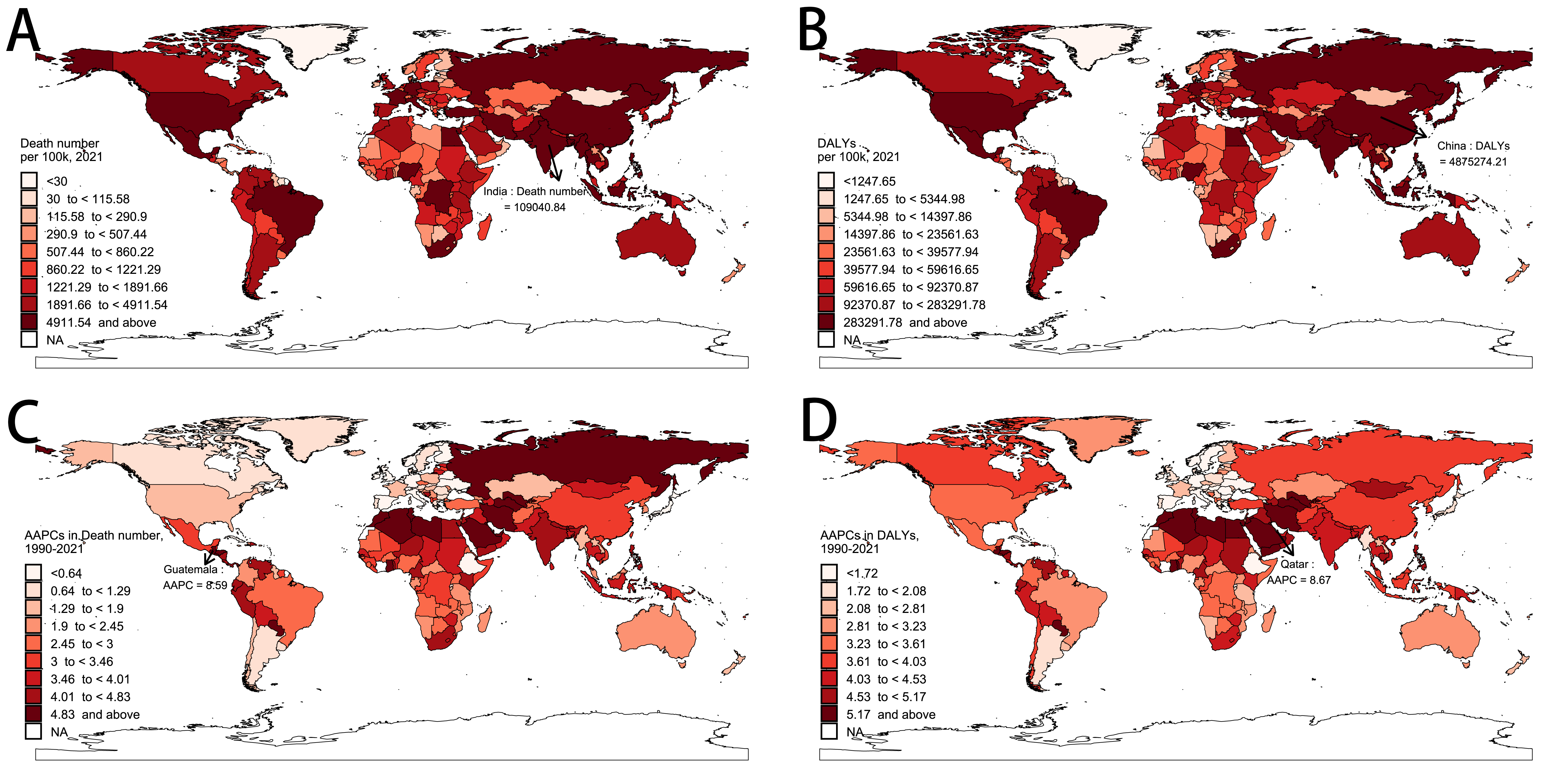


Figure S1. The Deaths numbers and DALYs of T2D attributable to behavioral risks in 1990 and 2021 at the global and regional level, and their AAPCs from 1990 to 2021. (A) Deaths numbers; (B) DALYs; (C) AAPCs in Deaths numbers; (D) AAPCs in DALYs. *DALYs,* disability-adjusted life-years; *AAPCs,* Average Annual Percentage Changes (Global Burden of Diseases Study, 204 countries or territories, 2021).


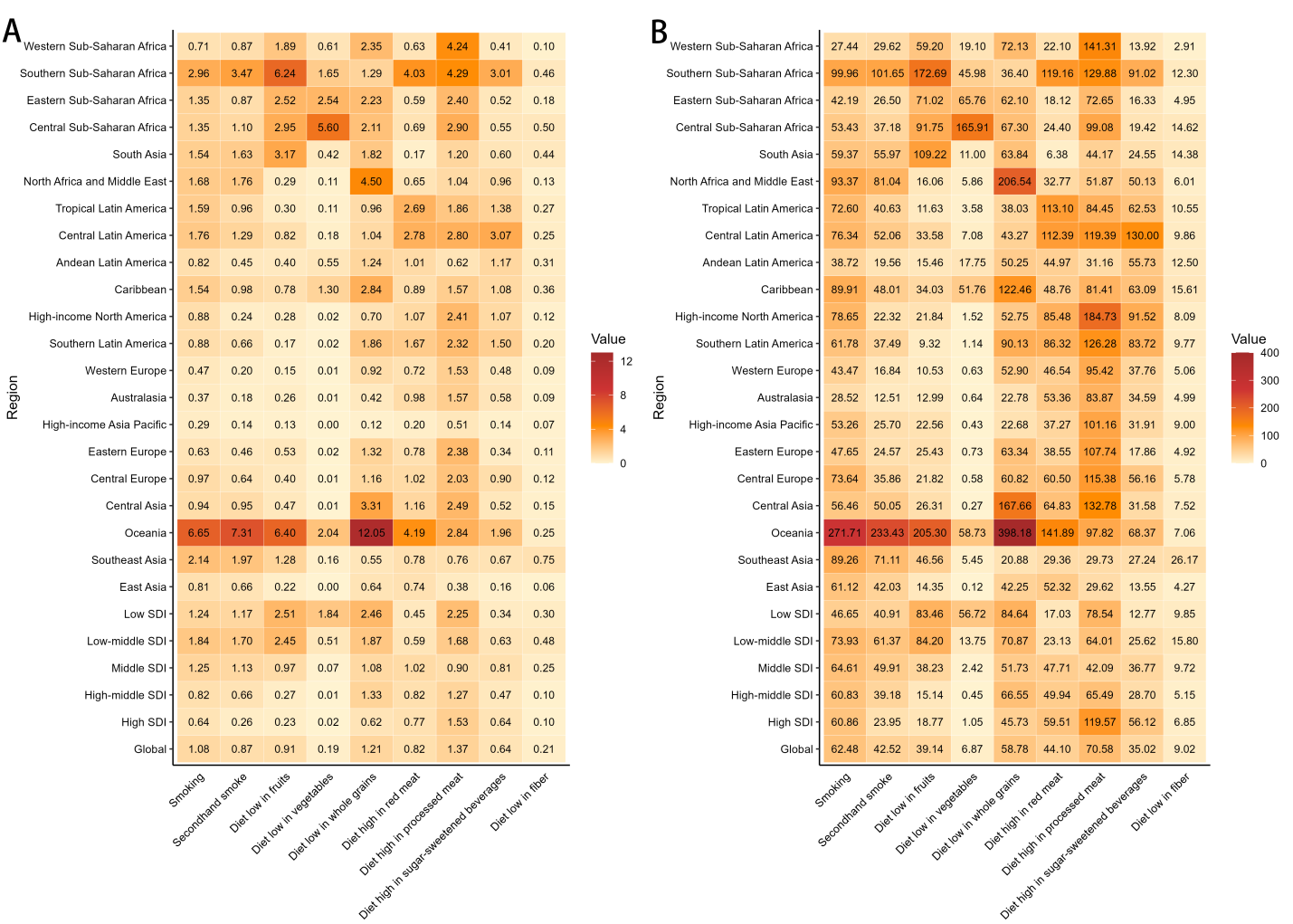


Figure S2. The ASMR and ASDR of T2D attributed to the secondary classification of four behavioral risks in 2021. (A) ASMR; (B) ASDR. (Global Burden of Diseases Study, 21 Global Burden of Diseases regions, 2021).


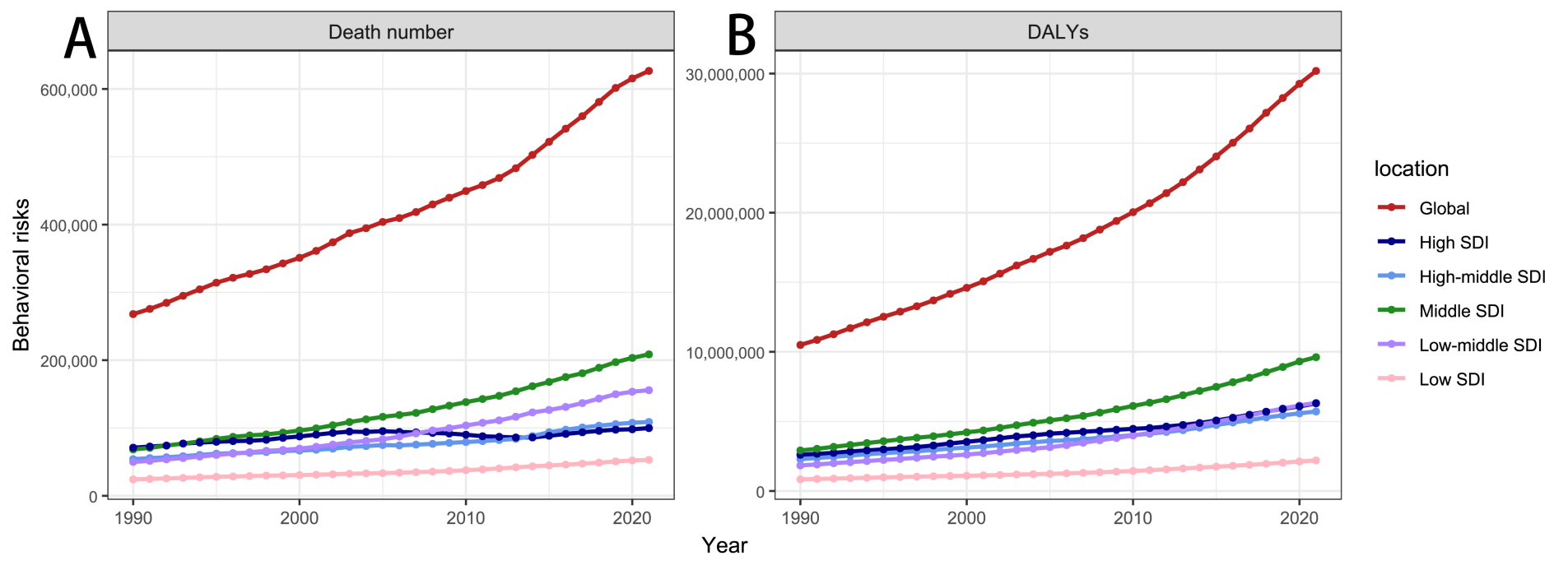


Figure S3. Trends in ASMR and ASDR of T2D attributable to behavioral risks globally and across SDI regions from 1990 to 2021. (A) Deaths numbers; (B) DALYs. *DALYs,* disability-adjusted life-years; *SDI,* socio-demographic index. (Global Burden of Diseases Study, 21 Global Burden of Diseases regions, 1990–2021).


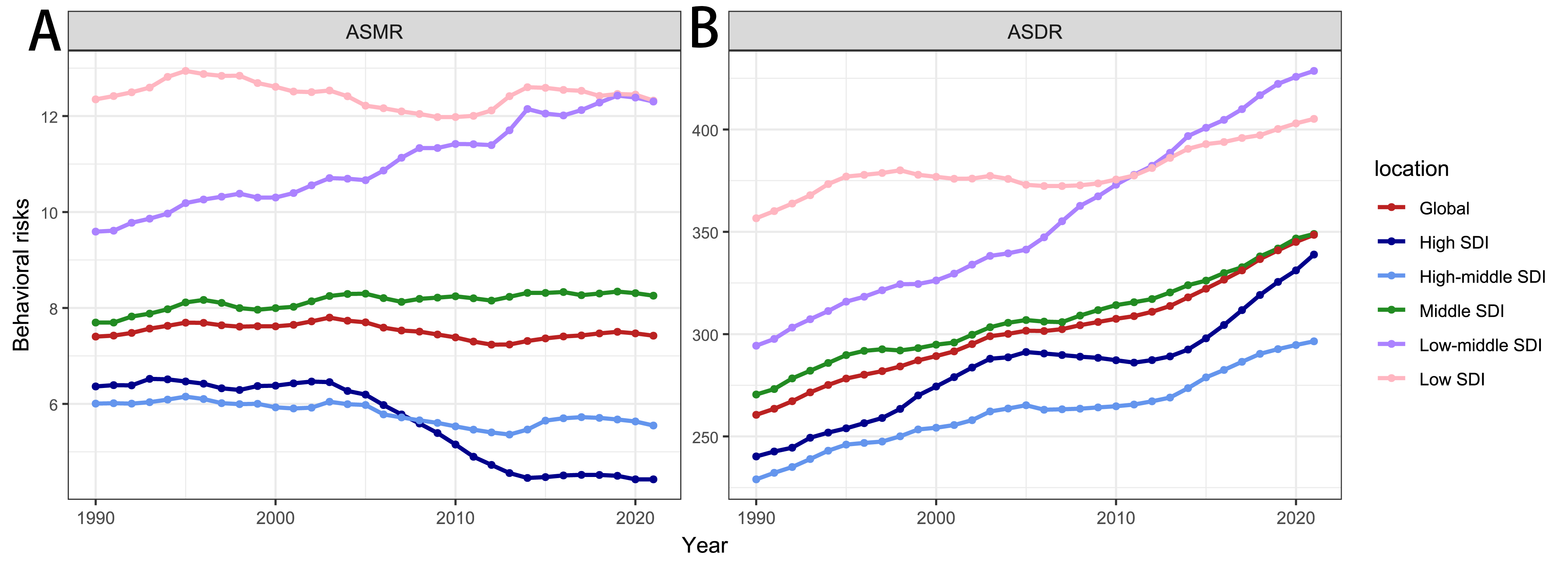


Figure S4. Trends in ASMR and ASDR of T2D attributable to behavioral risks globally and across SDI regions from 1990 to 2021. (A) ASMR; (B) ASDR. *T2D,* Type 2 Diabetes; *ASMR,* age-standardized mortality rate; *ASDR,* age-standardized DALY rate; *SDI,* socio-demographic index (Global Burden of Diseases Study, 21 Global Burden of Diseases regions, 1990–2021).


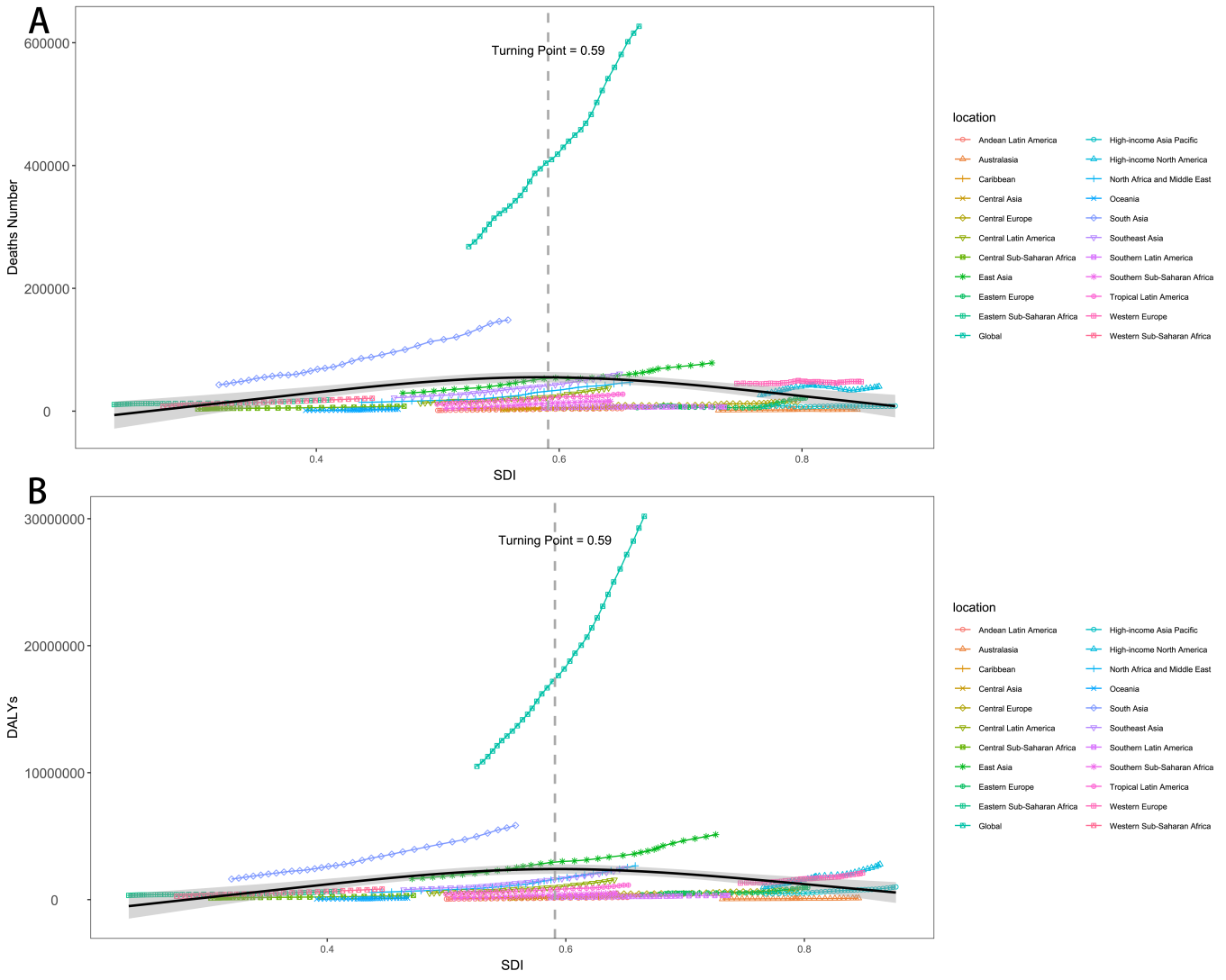


Figure S5. The Death numbers and DALYs of T2D attributable to behavioral risks, globally and for 21 GBD regions, by SDI from 1990 to 2021. (A) Deaths numbers; (B) DALYs. *T2D,* *DALYs,* Disability-Adjusted Life Years; *SDI,* socio-demographic index (Global Burden of Diseases Study, 21 Global Burden of Diseases regions, 2021).


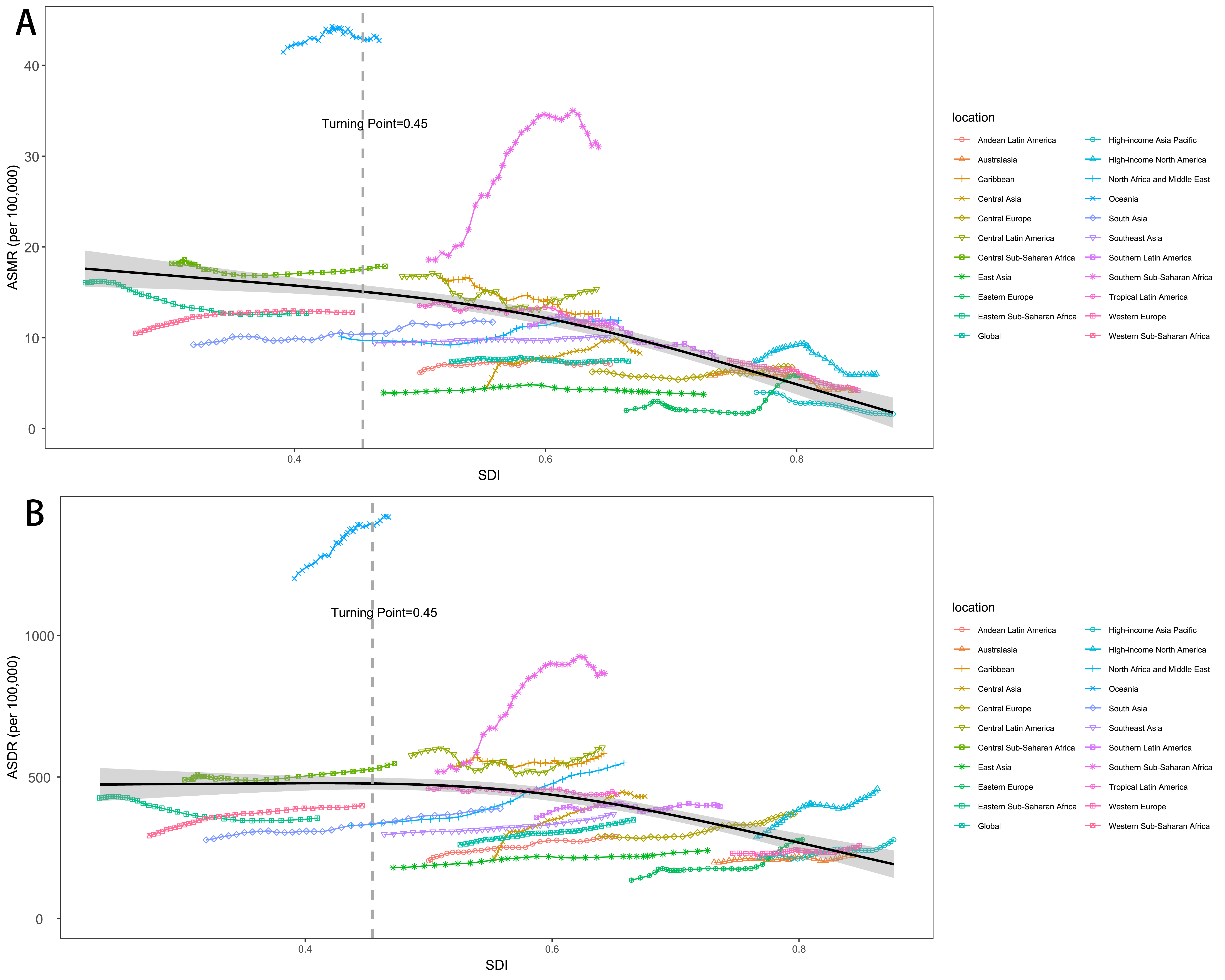


Figure S6. The ASMR and ASDR of T2D attributable to behavioral risks, globally and for 21 GBD regions, by SDI from 1990 to 2021. (A) ASMR; (B) ASDR. *ASMR,* age-standardized mortality rate; *ASDR,* age-standardized DALY rate; *SDI,* socio-demographic index (Global Burden of Diseases Study, 21 Global Burden of Diseases regions, 2021).


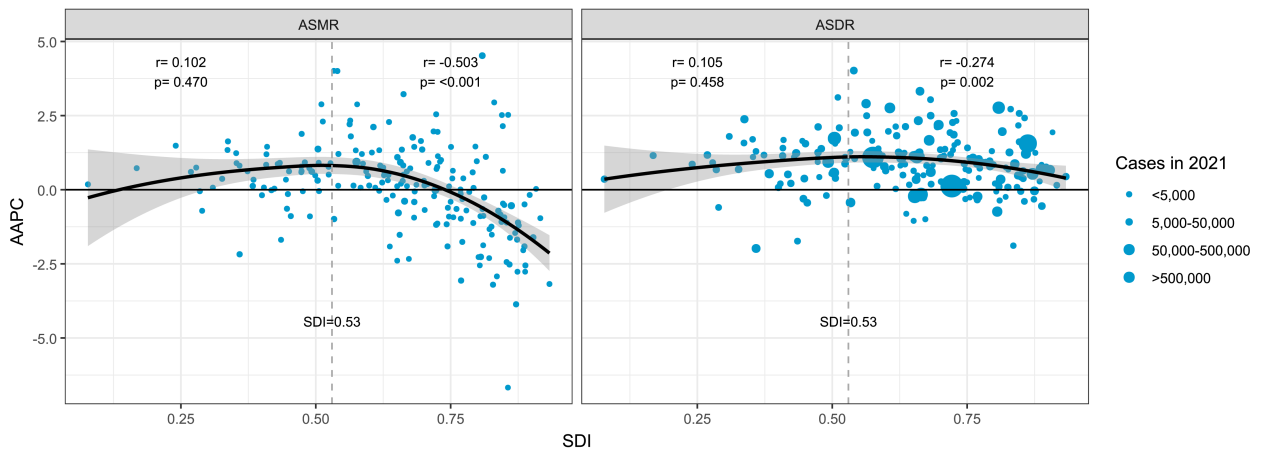


Figure S7. The relationship between AAPCs in ASMR and ASDR of T2D attributable to behavioral risks with SDI in 2021. (A) ASMR; (B) ASDR. *ASMR,* age-standardized mortality rate; *ASDR,* age-standardized DALY rate; *SDI,* socio-demographic index (Global Burden of Diseases Study, 204 countries or territories, 2021).


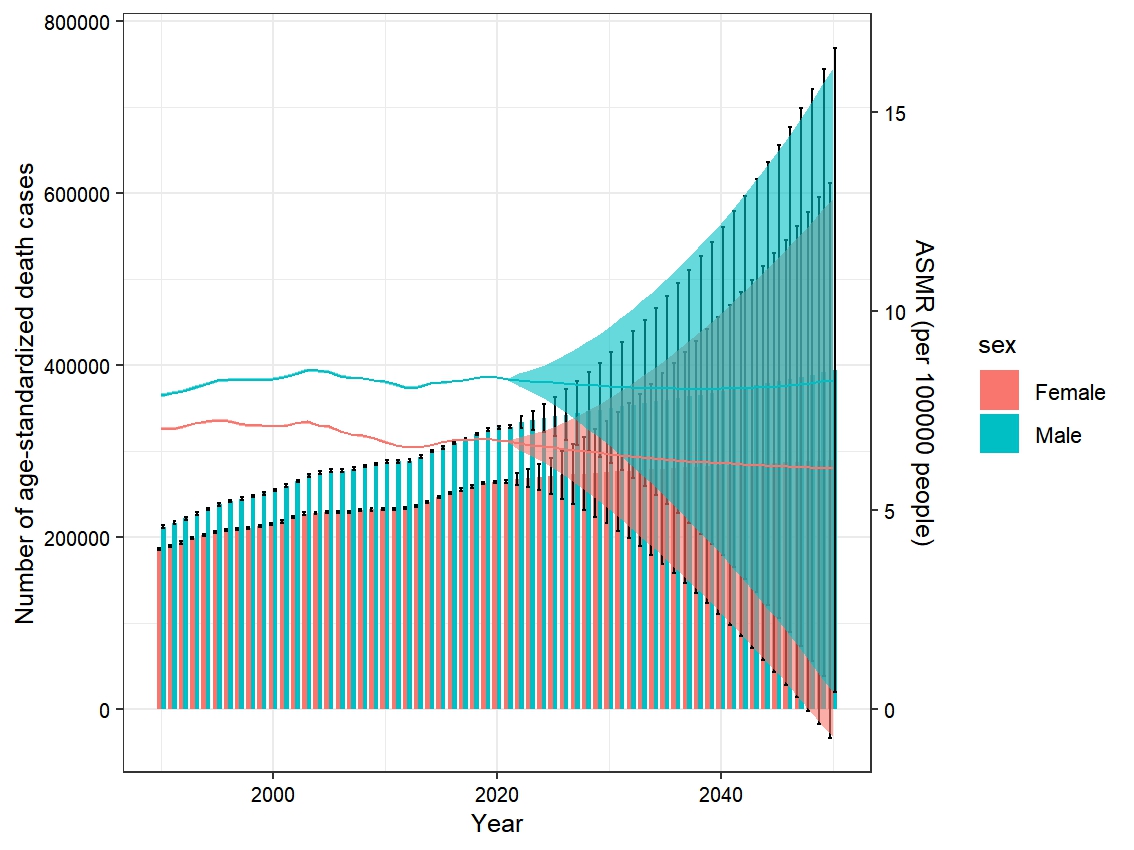
 **
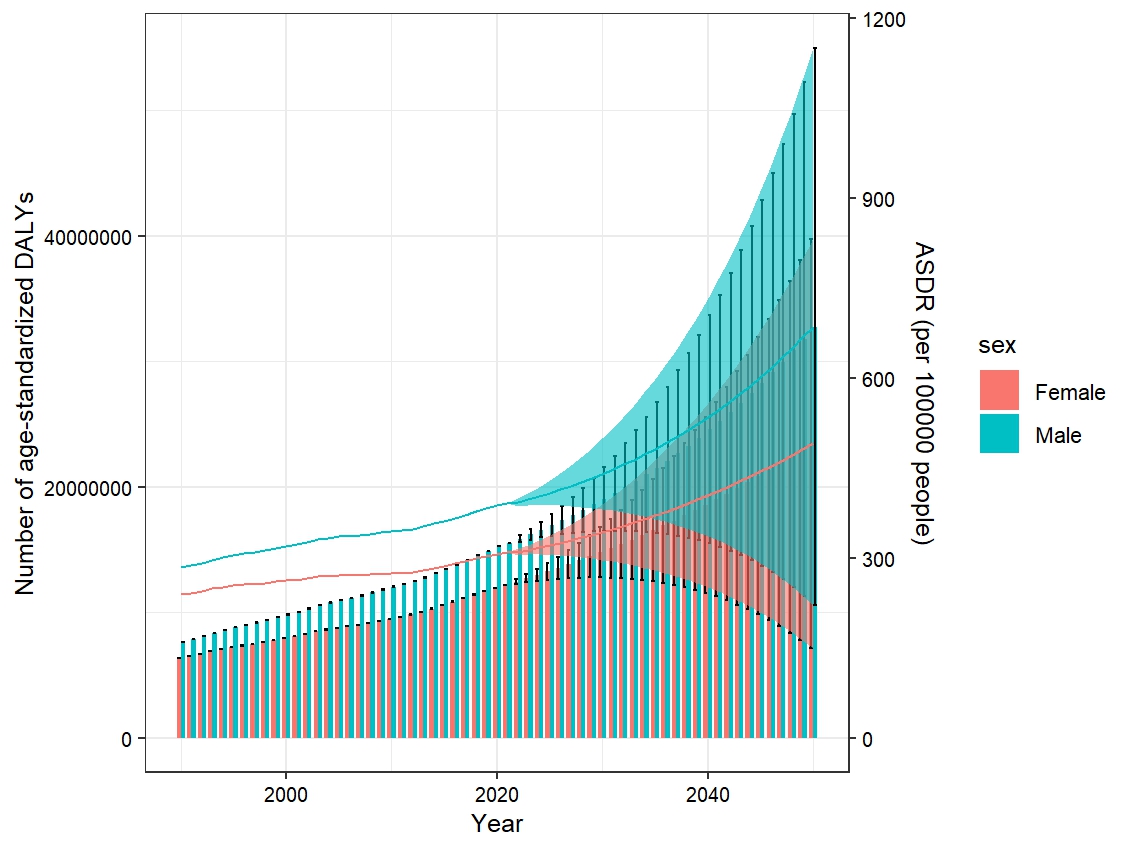
**


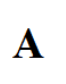

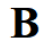


Figure S8. Temporal trends of the number and age-standardized rates for T2D attributable to behavioral risks at the global level and by sex from 1990 to 2050.

Deaths cases; (B) DALYs. *DALYs,* Disability-Adjusted Life Years; *ASMR,* age-standardized mortality rate; *ASDR,* age-standardized DALY rate (Global Burden of Diseases Study, 1990-2021).
